# Dissecting the effects of METTL3 on alternative splicing in prostate cancer

**DOI:** 10.3389/fonc.2023.1227016

**Published:** 2023-08-22

**Authors:** Lin Wang, Ling Shi, Yonghao Liang, Judy Kin-Wing Ng, Chan Hoi Yin, Lingyi Wang, Jinpao Hou, Yiwei Wang, Cathy Sin-Hang Fung, Peter Ka-Fung Chiu, Chi-Fai Ng, Stephen Kwok-Wing Tsui

**Affiliations:** ^1^ Metabolic Disease Research Center, Zhengzhou Central Hospital Affiliated to Zhengzhou University, Zhengzhou, China; ^2^ School of Biomedical Sciences, The Chinese University of Hong Kong, Hong Kong, Hong Kong SAR, China; ^3^ SH Ho Urology Centre, Department of Surgery, The Chinese University of Hong Kong, Hong Kong, Hong Kong SAR, China; ^4^ Hong Kong Bioinformatics Centre, The Chinese University of Hong Kong, Hong Kong, Hong Kong SAR, China

**Keywords:** METTL3, prostate cancer, RNA splicing, N 6 -methyladenosine, MKNK2, nanopore direct RNA sequencing

## Abstract

Although the role of METTL3 has been extensively studied in many cancers, its role in isoform switching in prostate cancer (PCa) has been poorly explored. To investigate its role, we applied standard RNA-sequencing and long-read direct RNA-sequencing from Oxford Nanopore to examine how METTL3 affects alternative splicing (AS) in two PCa cell lines. By dissecting genome-wide METTL3-regulated AS events, we noted that two PCa cell lines (representing two different PCa subtypes, androgen-sensitive or resistant) behave differently in exon skipping and intron retention events following METTL3 depletion, suggesting AS heterogeneity in PCa. Moreover, we revealed that METTL3-regulated AS is dependent on N^6^-methyladenosine (m^6^A) and distinct splicing factors. Analysis of the AS landscape also revealed cell type specific AS signatures for some genes (e.g., MKNK2) involved in key functions in PCa tumorigenesis. Finally, we also validated the clinical relevance of MKNK2 AS events in PCa patients and pointed to the possible regulatory mechanism related to m^6^A in the exon14a/b region and SRSF1. Overall, we characterize the role of METTL3 in regulating PCa-associated AS programs, expand the role of METTL3 in tumorigenesis, and suggest that MKNK2 AS events may serve as a new potential prognostic biomarker.

## 1 Introduction

PCa is the second most prevalent malignancy in males and the fifth leading cause of cancer mortality in men worldwide (1). Clinically, relatively indolent PCa (i.e., Gleason grade (GS)≤7) is treated with radical prostatectomy with a favorable prognosis, whereas locally progressed and metastatic PCa is typically treated with androgen deprivation therapy (ADT) (2). Notably, despite current therapies that improve prognosis, most patients will eventually fail ADT and progress to a deadly condition (androgen insensitive, AR^-^) known as castration-resistant PCa (CRPC) (3). The molecular mechanisms of PCa progression, particularly CRPC, remain largely undefined.

m^6^A modification is ubiquitous in most eukaryotes (4) and plays crucial roles in a variety of bioprocesses, such as DNA damage response (5). Analogous to DNA methylation, the dynamic m^6^A is regulated by the methyltransferase complex (writers), demethylases (erasers), and reader proteins (6). Notably, METTL3 catalyzes the m^6^A modification process, which can be dramatically reduced by METTL3 deletion (7). Recently, a growing number of studies have revealed the link between imbalanced m^6^A levels and cancer progression (6). Experimentally, METTL3 acts synergistically with YTHDF2 to inhibit the expression of SOCS2, encouraging liver cancer (HCC) development and progression (8). In PCa, METTL3 enhances MYC expression thus promoting cell proliferation (9), promotes the invasion through regulating the SHH-GLI1 signaling pathway (10), and modulates the HuR-ITGB1 signaling pathway thus contributing to bone metastasis (11). Despite a few findings in PCa concentrating on specific genes on a small scale, the potential biological consequence of global m^6^A abnormalities on a genome-wide scale remains deserved.

Additionally, m^6^A alternations not only influence gene expression by changing RNA stability and mRNA export but also regulate AS and 3′-end processing (12). AS is a dynamic process coupled with transcription and involved in many physiological functions as well as disease pathogenesis (13). Evidence has demonstrated that METTL3 deletion can attenuate the inflammatory response induced by lipopolysaccharide by modifying the expression patterns of MyD88 isoforms (14). Moreover, the m^6^A reader YTHDC1 can interact with SRSF3 to regulate exon inclusion (15), while FTO regulates the process of SRSF2 binding to the m^6^A-containing RUNX1T1 transcript and results in exon skipping (16). However, a comprehensive study of the AS landscape governed by METTL3 has not been conducted in PCa.

Recently, the development of long-read sequencing (e.g., direct RNA sequencing of Oxford Nanopore Technologies) provides the possibility to study splicing variants and alternative polyadenylation events (17), which directly obtain the structural information of the entire transcript without the assembling step like standard RNA sequencing (RNA-Seq). Another significant advantage of direct RNA sequencing (DRS) over standard RNA-Seq is the use of long reads to correctly dissect transcript isoforms and simultaneously detect RNA-modified sites (e.g., m^6^A) (18).

In this study, we combined standard RNA-Seq and long-read DRS of Oxford Nanopore to comprehensively dissect the impact of METTL3 on RNA splicing in the setting of two PCa cell lines with distinct AR sensitivities. We revealed that METTL3 potentially had shared and unique mechanisms affecting alternative splicing in AR^+^ and AR^-^ PCa cells. The different effects of METTL3 on exon skipping and intron retention might be related to distinct splicing partners.

## 2 Materials and methods

### 2.1 Cell culture

Normal epithelial prostate cell line (RWPE-1) and PCa cell lines (LNCaP and DU145) were obtained from Prof. Andrew Chan’s laboratory in the School of Biomedical Sciences, The Chinese University of Hong Kong. RWPE-1 cells were cultured using the KSFM-1 medium kit (Thermo Fisher, No.17005042) with supplementary bovine pituitary extract (BPE) and human recombinant epidermal growth factor (rEGF). LNCaP cells were maintained in RPMI-1640 medium (Invitrogen) supplemented with 10% FBS and 1% PS, and DU145 cells were cultured in DMEM (Invitrogen) supplemented with 10% FBS and 1% PS. All cells were incubated at 37°C and 5% CO2.

### 2.2 siRNA transfection, RNA extraction, and polyA RNA isolation

The siRNA against METTL3 (Invitrogen, Catalog: #4392422) or control was transfected into LNCaP and DU145 cells using RNAiMAX reagent (Invitrogen). Total RNA was extracted with a Pure-link mini kit (Invitrogen) 48 h after transfection. RNA concentration was measured via a Nanodrop 2000, and RNA integrity (RIN) was detected using an RNA 6000 nanochip (Agilent Technologies) (Requirement: RIN>8.8). PolyA RNA was isolated from 75 μg of total RNA using a Dynabeads™ kit.

### 2.3 Real-time quantitative PCR and western blot

cDNA transcription was performed using a SuperScript III synthesis kit (Invitrogen) and then used for qPCR based on SYBR (Applied Biosystems). Two siRNAs (siM3-1 and siM3-2) targeting METTL3 were designed, and we observed that the knockdown efficiency of siM3-1 was only 50%, which we considered insufficient for further analyses. Therefore, the more effective target-2 (siM3-2) was selected for the following analysis (**Figure S1**). The sequences of primers and siRNAs used in this study are shown in **Supplementary Table S1**.

The siRNA-transfected LNCaP and DU145 cells were lysed in RIPA lysis buffer and Western blotting was conducted as previously (9). Then, the intensity of METTL3 (Proteintech, 15073-1-AP) and GAPDH (Santa Cruz, sc-25778) bands was quantified using ImageJ software and GAPDH was used as the housekeeping marker.

### 2.4 RNA-Seq data processing

Paired-end sequencing (2 × 150 bp) of siControl- and siMETTL3-transfected cells was performed on a HiSeq 4000 sequencing platform (Illumina). Clean reads were aligned to the human hg38 genome (UCSC) using HISAT2 (v2.2.1) software (19). PCA of three replicates was executed, with one outlier in the DU145 siControl group excluded from downstream analysis. Differential gene expression (DEG) analysis was conducted using DESeq2 (20). Gene Ontology (GO) and KEGG enrichment analyses were performed using the clusterProfiler R package (21).

### 2.5 TCGA data analysis

A table containing FPKM values from 551 adjacent normal, and PCa tissue samples was downloaded from the TCGA database (https://portal.gdc.cancer.gov/projects/TCGA-PRAD), wherein we also retrieved the clinical data of 499 primary PCa patients. This FPKM table was used for expression profile analysis of m^6^A regulators. The association between METTL3 expression and overall survival and disease-free survival was determined using the GEPIA2 web server (22). The functional enrichment of m^6^A regulators was analyzed using Metascape (23) and displayed using Cytoscape software (24).

### 2.6 Nanopore DRS data processing

As previously described, each 1 μg purified polyA RNA was subjected to library preparation following the SQK_RNA002 kit procedure and consequently sequenced on a Nanopore GridION platform for a 48-h runtime (25). Base-calling was performed with Guppy (v4.2.2) to generate passed reads with quality scores ≥7. The alignment of passed reads against human hg38 (UCSC) and transcriptome (GENCODE v32) was implemented via minimap2 (v2.17-r941) (26). Statistics of the passed reads were assessed using NanoStat (v1.4.0) (27). The sequence length distribution of each sample was generated using the wub package (https://github.com/Nanoporetech/wub).

### 2.7 AS analysis of DRS and RNA-Seq data

Splicing analysis of DRS data was performed with the FLAIR (v1.5) pipeline (17). Specifically, BED12 files were converted from primary BAM files and then corrected by annotated GTF files and junction files. Junction files from RNA-Seq data were obtained from the same cell lines, and splice sites (< 3 reads) were filtered out. Then, the corrected files were assembled into high-confidence isoforms with the stringent parameters of at least three supporting reads. Splicing events were determined using the FLAIR diffSplice module based on Fisher’s exact test.

Differential AS events of RNA-Seq data were identified using rMATS (v4.1.0) with a strict parameter of cstat=0.05 (28, 29) and filtered with a p-value <0.05. As an indicator of splicing events, the percent spliced in (PSI) index of differential intron retention and exon splicing events refers to the ratio of included reads to total reads (sum of included and excluded reads) (30). The PSI values of differential AS events were assessed using the Wilcoxon rank-sum test, which is used to provide a global overview of inclusion/exclusion levels between the control and knockdown groups. The global difference in PSI distributions was also evaluated by using FDR<0.05, which was supplemented in the **Supplementary Figure S8**.

The PSI index of MKNK2a/b isoforms in PCa was downloaded from the TCGA SpliceSeq database. The MKNK2_AA_46570 event represents the PSI index of sequencing reads covering between exon 13 (e13) and exon 14a (e14a), indicating the expression of the MKNK2a isoform. While the MKNK2_AT_46567 represents the PSI index of the sequencing reads covering between exon 13 and exon 14b (e14b), producing the MKNK2b isoform. According to the previous reference (31), we set the threshold at the 80^th^ percentile, dividing the PCa patients into high PSI and low PSI groups. The survival difference between high and low PSI groups was calculated by using “survival” (3.5) and “survminer” (0.4.9) R software.

### 2.8 Detection of differential m^6^A sites

Differential m^6^A sites between the siControl and siMETTL3 groups were detected using ELIGOS2 and DRUMMER (32, 33). Both approaches were designed to identify RNA modifications through the comparative analysis of base-calling errors in the Nanopore DRS dataset (34). In our study, ELIGOS2 was utilized to assess the global difference in m^6^A modifications, employing strict threshold values of p<0.05 and odd ratio >1.2 (https://gitlab.com/piroonj/eligos2) (32). The criterion of “odd ratio >1.2” indicated at least a 1.2-fold difference between the control and METTL3 knockdown conditions. Therefore, the differential m^6^A sites utilized in the further analysis primarily represented the decreased m^6^A sites upon METTL3 knockdown. DRUMMER can identify RNA modifications at each site on distinct transcript isoforms by using isoform mode (https://github.com/DepledgeLab/DRUMMER) (33). Therefore, DRUMMER was utilized to detect isoform-specific differences in m^6^A levels. All differential m^6^A data from ELIGOS2 and DRUMMER were then selected with a p-value <0.05 and the reference “A” site. The 5-kmer motif of differential m^6^A sites was generated *via* WebLogo (https://weblogo.berkeley.edu/logo.cgi). The genomic features of m^6^A sites were annotated with the ChIPseeker R package (35). The detailed differential m^6^A results were included in the **Supplementary Tables S3–6**.

### 2.9 Motif analysis of spliced genes

The regions of differential intron retention and exon skipping events were extracted for motif analysis using HOMER (http://homer.ucsd.edu/homer/motif/) software. The resulting motifs were visualized using the ggseqlogo R package.

### 2.10 Statistical analysis

Differences in PSI values of differential intron retention and exon skipping events between groups were assessed using the Wilcoxon rank-sum test. The significance of overlaps between m^6^A sites and DEGs, spliced genes, or junction regions was determined using a hypergeometric test by R software. The significance of candidate genes in qPCR experiments was determined by T-test.

## 3 Results

### 3.1 Increased expression of METTL3 was associated with an aggressive PCa status

**Figure 1:**
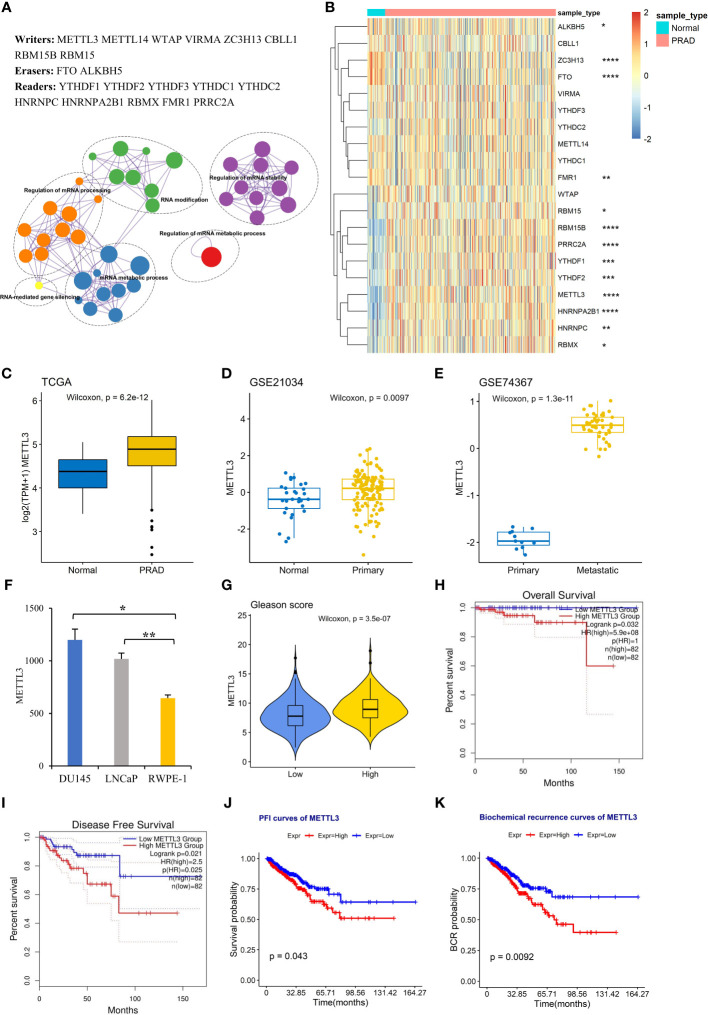
High METTL3 expression is associated with an aggressive PCa status. **(A)** Genes and functional ontology related to m^6^A. **(B)** Heatmap showing the expression profile of 17 m^6^A regulators in adjacent normal (n=52) and PRAD tissues (n=499). The expression level of METTL3 was analyzed in **(C)** TCGA PRAD dataset, **(D)** GSE21034, **(E)** GSE74367, and **(F)** PCa cell lines (GSE35401). The error bars indicate the variation between the biological replicates in each dataset. **(G)** The difference in METTL3 expression between the low Gleason score (≤7) and high Gleason score (>7) groups. Overall survival **(H)**, disease-free survival **(I)**, progression-free interval **(J)**, and biochemical recurrence **(K)** curves for the high and low METTL3 groups based on the median values. *p<0.05; **p<0.01; ***p <0.001; ****p <0.0001.

Functional enrichment indicated that m^6^A regulators were more associated with mRNA stability and mRNA processing regulation (**Figure 1A**). To investigate how m^6^A regulates mRNA processing in PCa, we first conducted an analysis of the expression profiles of m^6^A regulators by retrieving the FPKM expression data from the TCGA database, comprising adjacent normal (n=52) and prostate adenocarcinoma (n=499). Most m^6^A “writer” and “reader” proteins were overexpressed in PCa tissues compared to normal tissues (**Figure 1B**), indicating high m^6^A levels in PCa.

In particular, we focused on METTL3, the catalytic submit for m^6^A modifications (7), and its expression patterns in various stages of PCa. We observed that the increase in METTL3 expression between normal and primary groups was statically significant but relatively modest in both TCGA and GSE21034 datasets (**Figures 1C, D**). However, a more substantial change in METTL3 was evident when comparing the primary and metastatic groups (GSE74367) (**Figure 1E**), supposing a potential role of METTL3 in PCa metastasis. The overexpression of METTL3 was also observed in PCa cell lines LNCaP (androgen-sensitive, AR^+^) and DU145 (androgen insensitive, AR^-^) compared to normal prostate cells (RWPE-1) (**Figure 1F**, GSE35401) (36). To explore the clinical significance of METTL3 in PCa, we found that the higher METTL3 level was positively associated with a high Gleason score (>7) (**Figure 1G**). Furthermore, overrepresented METTL3 was linked to aggressive survival stages, including overall survival (OS), disease-free survival (DFS), progression-free interval (PFI), and biochemical recurrence (**Figures 1H–K**). Collectively, these findings suggested a pro-oncogenic role of METTL3 in PCa progression.

### 3.2 Elucidating functional pathways influenced by METTL3 depletion

**Figure 2:**
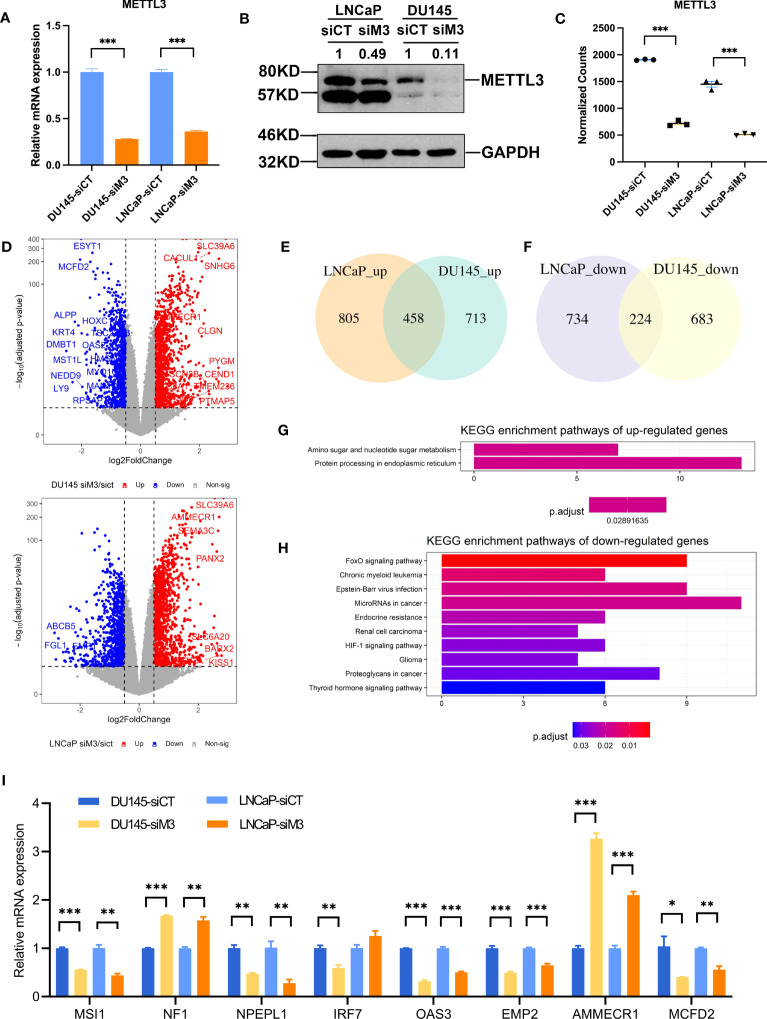
Functional annotation of DEGs of the two PCa cell lines. **(A, B)** The relative mRNA and protein level of METTL3 in siControl and siMETTL3 groups of LNCaP and DU145 cells. **(C)** The normalized counts of METTL3 in siControl and siMETTL3 groups of RNA-Seq data. **(D)** Volcano plot of DEGs in the comparison of the siMETTL3 against siControl groups of DU145 and LNCaP cells. The X-axis shows log2fold changes in expression and the Y-axis represents –log10 (adjusted p-value). DEGs with a padj < 0.05 & abs|log2FC| >0.5 were considered significantly differentially expressed. **(E, F)** Venn plot indicated the overlapped genes of upregulated and downregulated DEGs of the two PCa cells. **(G, H)** KEGG functional annotation of overlapped upregulated or downregulated genes. **(I)** The relative mRNA levels of candidate genes in the two cell lines upon METTL3 depletion. siCT, siControl; siM3, siMETTL3; *p<0.05; **p<0.01; ***p<0.001.

To gain further insight into the molecular mechanism by METTL3 in PCa, we performed RNA-Seq in DU145 (AR^-^) and LNCaP (AR^+^) cells with METTL3 knockdown. First, the knockdown efficiency of siRNA against METTL3 was assessed in the two PCa cell lines by qPCR and western blotting and shown in **Figures 2A, B**. In parallel with qPCR and western blotting assessment, the successful knockdown of METTL3 was also confirmed by RNA-Seq data (**Figure 2C**). Subsequently, we evaluated genes potentially regulated by METTL3 using DESeq2. It was found that 2078 DEGs in DU145 cells between siMETTL3 and siControl groups, of which 1171 genes were upregulated and 907 genes were downregulated (padj <0.05, **Figure 2D**). In LNCaP cells, we found 1,263 increased genes and 958 decreased genes along with METTL3 knockdown (padj <0.05, **Figure 2D**). The data revealed that METTL3 depletion has a global impact on gene expression.

We next explored the consistency between LNCaP and DU145 cells in response to METTL3 knockdown. There were 458 genes commonly upregulated, and 224 genes downregulated, respectively (**Figures 2E, F**). KEGG enrichment of these DEGs indicated that METTL3 was negatively associated with protein processing in endoplasmic reticulum, while favorably connected to various cancer-related pathways, such as HIF-1 signaling, endocrine resistance, FoxO signaling, and proteoglycans (**Figures 2G, H**). We selected eight known cancer driver genes and PCa-survival genes (37, 38) and then validated the expression change using qPCR. As a result, the expression changes of these eight METTL3-regulated genes showed a consistent trend with RNA-Seq data, demonstrating the accuracy of our RNA-Seq data (**Figure 2I**). Moreover, these eight genes were identified as having at least one annotated m^6^A site in public miCLIP data (39, 40) (**Figure S2**), supposing the potential regulation by m^6^A. However, more research is required to determine the potential mechanisms involving these genes (e.g., MSI1, NPEPL1).

More interestingly, GSVA analysis also showed that many cancer hallmarks were dysregulated between siControl and siMETTL3 groups, with a noticeable unique pattern in LNCaP (AR^+^) and DU145 (AR^-^) cells (**Figures S3A, C**). For instance, immune (e.g., IFN gamma/alpha response) and metabolism-related (e.g., cholesterol and adipogenesis) signatures were less active in the siMETTL3 group of DU145 cells (**Figure S3B**). In contrast, metabolism pathways such as cholesterol homeostasis and adipogenesis were more activated in the siMETTL3 LNCaP group (**Figure S3D**). This may be relevant to cell-specific genomic properties and AR dependence. Previous studies have demonstrated that AR controls critical genes involved in glucose metabolism and lipid metabolism (41). Taken together, the data imply that METTL3 may be engaged in multiple common cancer-related pathways, thus performing pro-oncogenic activities in PCa cells. On the other hand, METTL3 may have partially dissimilar functions in AR^+^ and AR^-^ PCa cells, especially in terms of metabolism.

### 3.3 Depletion of METTL3 impacts AS landscape

To gain an insight into the role of METTL3 in AS regulation, we applied direct RNA-sequencing (DRS) performed using the Oxford Nanopore long-read sequencing platform to generate METTL3-dependent AS profiles in LNCaP (AR^+^) and DU145 (AR^-^) cells. Overall, 0.6-1.5 million reads were generated for each sample, with an average read length of 1.0 kb and average read quality of 11 (**Figure S4**; **Table S2**). Approximately 95% and 99% of the passed reads were aligned to the human genome hg38 and transcriptome (GENCODE v32), respectively (**Table S2**).

**Figure 3:**
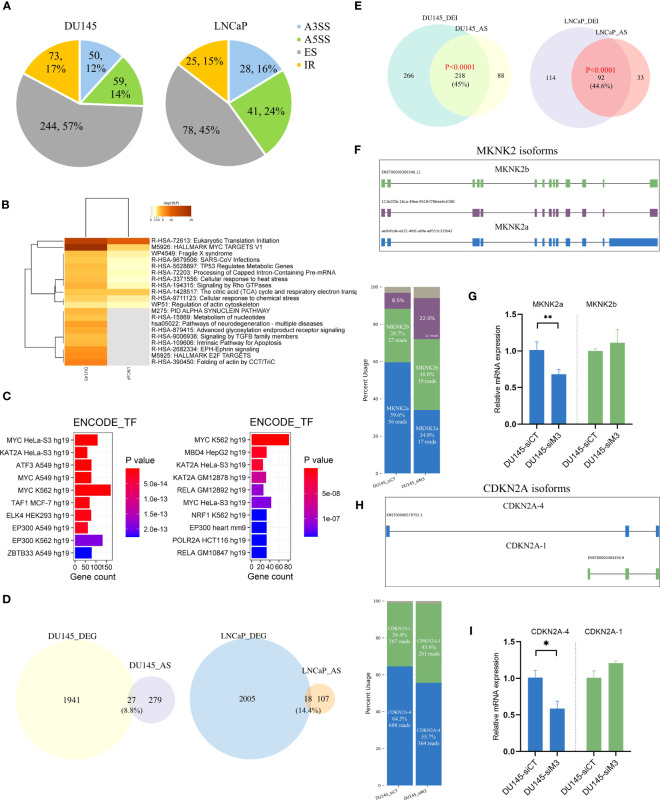
The global overview of differential AS events in DU145 and LNCaP cells. **(A)** Pie chart of differential AS events between siControl and siMETTL3 groups of DU145 and LNCaP cells. **(B)** The top 20 functional annotations and **(C)** ENCODE TF terms of AS-associated genes in DU145 (left) and LNCaP (right) cells. **(D)** Venn plot showing the overlapped genes between DEGs and AS-related genes in DU145 and LNCaP cells. **(E)** The intersection between differentially expressed isoforms (DEI) and AS harboring genes. Significance was assessed by a hypergeometric test. **(F, H)** Schematic diagram of isoforms of two known spliced genes (MKNK2 and CDKN2A) in DU145 or LNCaP cells. Stacked bar plots showing the relative proportion and the corresponding read count of each isoform and isoforms were labeled with different colors. **(G, I)** Bar plots displayed the relative mRNA expression of two main isoforms of MKNK2 and CDKN2A genes. siCT, siControl; siM3, siMETTL3. *p<0.05; **p<0.01.

Then, we used FLAIR (17) software to identify the differential AS events upon METTL3 knockdown. Four types of AS events were obtained, including exon skipping (ES), alternative 5’/3’-splice site (A5SS/A3SS), and intron retention (IR). A total of 426 and 172 differential AS events were identified in DU145 and LNCaP cells, respectively (**Figure 3A**). The predominant AS type in the two cells was the ES type.

The functional enrichment revealed that differential AS events were mainly associated with eukaryotic translation initiation, cellular response to stimuli (e.g., heat, chemical), metabolic pathways, and proliferation hallmarks (MYC targets v1, E2F targets) (**Figure 3B**). Additionally, transcription factor (TF) analysis showed that differential AS events were highly associated with MYC (**Figure 3C**), supposing the potential role of MYC in AS regulation.

### 3.4 METTL3 regulates isoforms switching

As we know that AS is usually coupled with transcriptional processes and thus influences gene expression (42). We found that the functional annotation of METTL3-regulated spliced genes was in line with those discovered in DEGs. However, when intersected AS genes with previously identified DEGs, we noted that only 9-15% of AS genes overlapped (**Figure 3D**), suggesting that AS dysregulation may have a minor influence on global gene expression but rather functionally tuned transcriptomes (43).

Then, using FLAIR (17), we inspected the isoform-level profiles to explore the functional implications of AS dysregulation on PCa transcriptome. We discovered that, as expected, the majority of differentially expressed isoforms (DEIs) were generated due to aberrant splicing events (**Figure 3E**). For example, two known spliced genes (e.g., MKNK2 and CDKN2A) were selected to show distinctive isoforms switching with METTL3 knockdown (**Figures 3F–I**) (**Figure S5-6**). Three isoforms of the MKNK2 gene were observed, with a proportionate rise in the shorter transcript ENST00000309340 (MKNK2b) and a comparable reduction in the longer transcript (MKNK2a) upon METTL3 depletion (**Figure 3F**). We validated the isoform switching of MKNK2 using qPCR and found a similar reduction of MKNK2a with METTL3 knockdown (**Figure 3G**). Another alternatively spliced gene, CDKN2A, has been found to express two major isoforms, of which the CDKN2A-4 isoform (ENST00000579755) was relatively decreased under the DU145 siMETTL3 condition (**Figure 3H**) (**Figure S5**). The qPCR experiment also verified a similar decrease in CDKN2A-4 and a slight rise in the CDKN2A-1 isoform upon METTL3 depletion (**Figure 3I**). However, more research has to be done to determine the precise mechanisms of these isoforms and how they relate to PCa development. Nevertheless, our findings suggest that METTL3-induced splicing abnormalities may influence PCa biology in part via isoform switching of key genes.

### 3.5 METTL3 regulates intron retention and exon-skipping events

We previously found that LNCaP (AR^+^) and DU145 (AR^-^) cells have different functional enrichments upon METTL3 depletion (**Figure S3**). Therefore, we further investigated the overlap of AS-related genes in the two PCa cells to see if they also with divergent phenotypes. As a result, there was only limited overlap, indicating cell specificity (**Figure S7**). This is to be expected, given the transcriptome changes drastically as the disease progresses.

**Figure 4:**
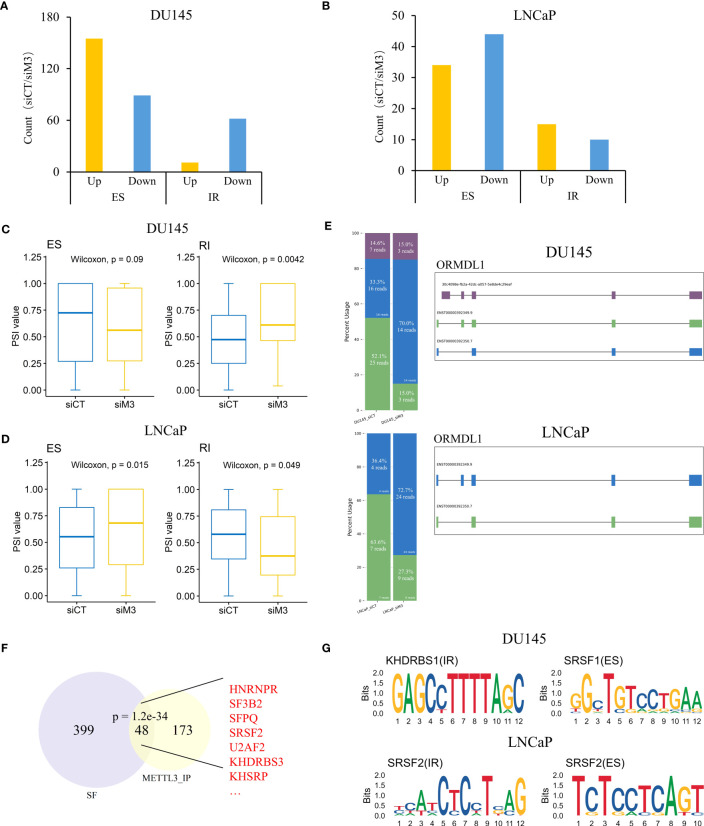
Depletion of METTL3 influences intron retention and exon skipping events in DU145 and LNCaP cells. **(A, B)** The bar plot of up and downregulated AS events shows a significant difference between siControl and siMETTL3 groups of DU145 and LNCaP cells. **(C, D)** Box plots showing PSI values of differential IR and ES events in DU145 and LNCaP cells, respectively. **(E)** Schematic diagram of isoforms from ORDML1 gene in DU145 or LNCaP cells. Stacked bar plots showing the relative proportion and the corresponding read count of each isoform and isoforms were labeled with different colors. **(F)** The intersection between METTL3 pulled-down proteins and known SFs. Significance was assessed by a hypergeometric test. Several typical intersecting genes were marked in red. **(G)** Predicted sequence motifs of differential IR and ES events in DU145 and LNCaP cells. siCT, siControl; siM3, siMETTL3; SFs, splicing factors.

Further, we analyzed the different effects of METTL3 on AS events in DU145 and LNCaP cells and we mainly focused on ES and IR types. In the comparison of siControl versus siMETTL3 of DU145 cells, we observed a preferential increase in upregulated ES (155 up/89 down) and downregulated IR events (11 up/62 down) (**Figure 4A**). On the other hand, METTL3 knockdown led to the opposite effect in LNCaP cells. That is fewer upregulated ES events (34 up/44 down) and downregulated IR events (15 up/10 down) (**Figure 4B**). To corroborate this finding, we measured the inclusion levels (PSI values) of differential IR and ES events between siControl and siMETTL3 groups using RNA-Seq data. Consistently, the opposing changes in PSI values of IR and ES events were found in DU145 and LNCaP cells (**Figures 4C, D**; **Figure S8**). This indicated an increase in retained introns and skipped exons in the DU145 siMETTL3 group, whereas the LNCaP siMETTL3 group had the opposite tendency. For instance, the transcript (ENST00000392350) of the ORMDL1 gene with exon 2 skipping showed an increased level in the DU145 siMETTL3 group but decreased in the LNCaP siMETTL3 group (**Figure 4E**; **Figure S9**). ORMDL1 has been reported to be associated with ceramide biosynthesis and as a regulator of sphingolipid levels implicated in cell proliferation as well as migration (44).

RNA splicing is a complicated process that is coregulated by multiple factors, including splice sites, cis-elements, and splicing factors (SFs) (42). Next, we intended to explore whether the distinct influence on IR and ES in AR^+^ and AR^-^ cells is associated with SFs. By analyzing the proteomic data of METTL3 reported by Yue et al. (45), we discovered a substantial overlap between METTL3 pulled-down proteins and known SFs (e.g., SRSF2, U2AF2, KHDRBS3, KHSRP) (P=1.2e-34, hypergeometric test), suggesting a possible interaction between METTL3 and SFs (**Figure 4F**). Furthermore, motif analysis revealed that SRSF1 (P=1e-13) and KHRBSH1 (P=1e-14) were enriched in differential ES and IR events of DU145 cells, whereas the SRSF2 (P=1e-8) motif was detected in differential ES and IR events for LNCaP cells (**Figure 4G**), indicating that different mechanisms may be involved in the two PCa cell lines. Taken together, we found that METTL3 depletion resulted in divergent phenotypes for IR and ES events in DU145 and LNCaP cells, and the regulatory effects of METTL3 may be associated with different SF proteins.

### 3.6 Differential m^6^A profiles

**Figure 5:**
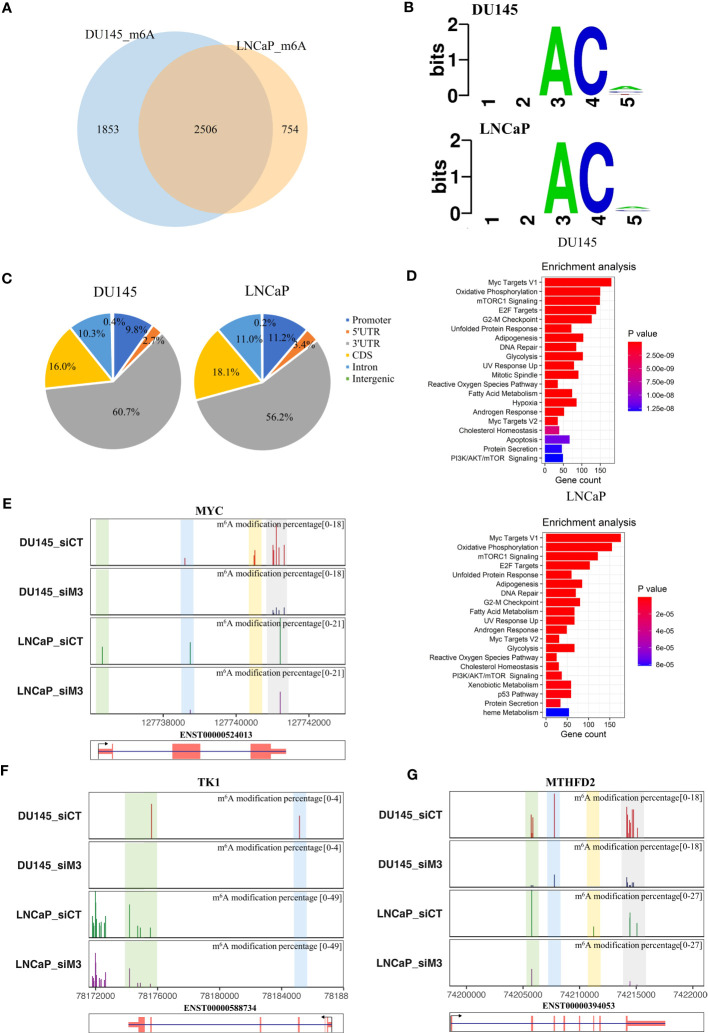
Decreased m^6^A profiles in DU145 and LNCaP cells with METTL3 depletion. **(A)** Venn diagram of the overlapping m^6^A-modified genes between DU145 and LNCaP cells. **(B)** The 5-kmer motif of differential m^6^A sites in DU145 and LNCaP cells after METTL3 knockdown. **(C)** The genomic feature distributions of decreased m^6^A sites in DU145 and LNCaP cells. **(D)** Cancer hallmark enrichments of m^6^A modified genes in DU145 and LNCaP cells, respectively. Only significant items with a p-value <0.05 are shown in the plot. **(E–G)** Examples of predicted m^6^A levels in MYC, TK1, and MTHFD2 genes. The top panel represented the proportion of predicted m^6^A levels in each gene under control (siCT) and METTL3 knockdown (siM3) condition in DU145 and LNCaP cells. Y-axis represents the m^6^A modification percentage, which is the percent error of specific bases (%ESB) identified by ELIGOS2 software. The bottom panel indicated the longest isoform of each gene.

To understand whether METTL3 modulates RNA splicing through m^6^A, we first analyzed differential m^6^A sites from DRS data using ELIGOS2 software with strict criteria, focusing primarily on decreased m^6^A sites (32)(seeing Methods). In DU145 and LNCaP cells, we found 15806 and 16940 potentially decreased m^6^A sites, respectively, with 2506 m^6^A-modified genes shared by the two cell lines (**Figure 5A**; **Tables S3, 4**). Moreover, the core residues of the 5-kmer motifs around these m^6^A sites were AC (**Figure 5B**), which was also consistent with prior results (46). Aside from the previously reported major enrichment in the 3’UTR, we observed that identified m^6^A sites in PCa cells partially fell into exonic (16.0% or 18.1%) and intronic regions (10.3% or 11%), indicating the potential involvement of m^6^A in IR and ES events (**Figure 5C**). Further, cancer hallmark enrichment of m^6^A-modified genes revealed a high association with “Myc target V1/V2”, “mTORC1 signaling”, and several metabolic pathways (**Figure 5D**). Remarkably, this finding also coincided with previous enrichment by altered genes or AS events.

We next investigated the relationship between decreased m^6^A sites and previously identified DEGs or splicing events. As a result, there were 443 (22.5%) and 280 (13.8%) overlapping genes between DEGs and m^6^A-modified genes in DU145 and LNCaP cells, respectively (P=1.87e-107 and P=2.76e-48, hypergeometric test) (**Figure S10A**). Additionally, we observed that 182 of 235 (77.4%) spliced genes in DU145 cells and 58 out of 79 (73.4%) genes in LNCaP cells intersected with m^6^A-modified genes (P=1.08e-158 and P=3.65e-56, hypergeometric test) (**Figure S10B**). The predicted m^6^A levels of three selected examples, namely MYC, TK1, and MTHFD2 genes were presented in **Figures 5E–G**. Notably, MYC has been previously shown to be regulated by METTL3 in PCa in an m^6^A-dependent manner (9). In addition, our analysis using the DRUMMER software also revealed intriguing isoform-specific differences in m^6^A levels (**Tables S5, 6**; **Figure S11**). These data suggest the function of METTL3 in the regulation of gene expression and RNA splicing in PCa cells might be mediated by m^6^A.

### 3.7 METTL3 modulates MKNK2 isoform expression by SRSF1 and m^6^A

**Figure 6:**
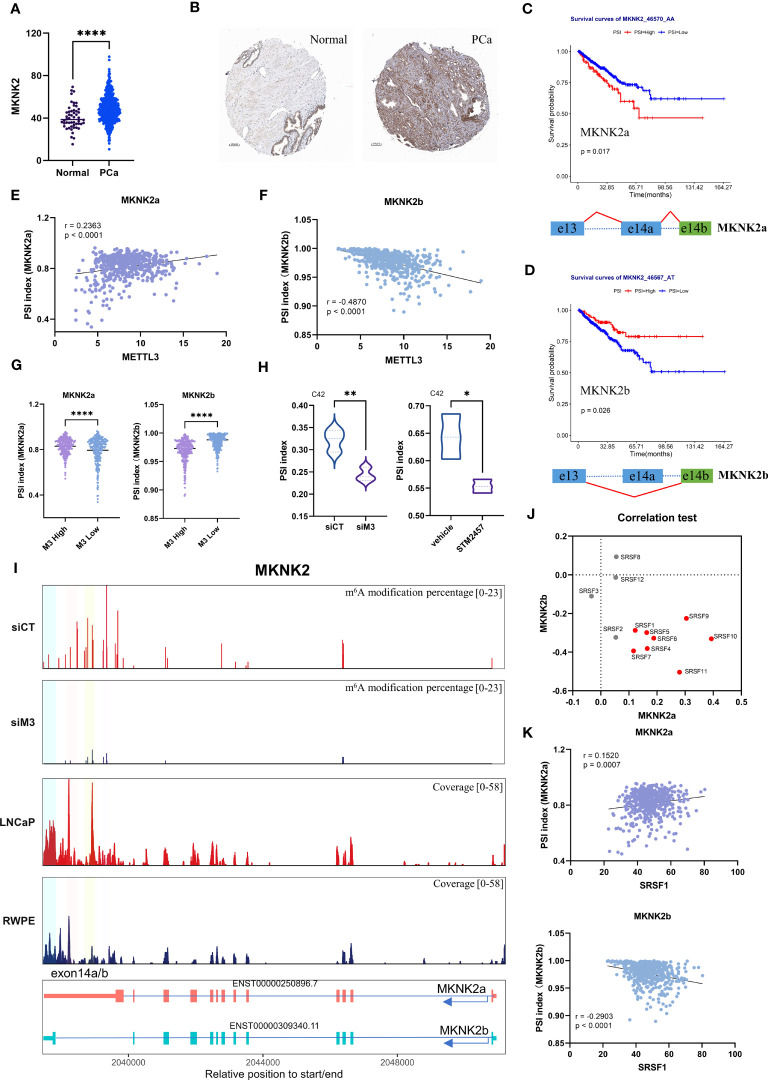
The potential mechanism of METTL3 in modulating MKNK2 splicing in PCa. **(A, B)** MKNK2 mRNA and protein levels in normal and PCa tissues. The immunohistochemical results were obtained from the human protein atlas. **(C, D)** Survival analysis showed a significant positive/negative correlation between MKNK2a/b isoforms and progression-free survival (PFI) in PCa patients. Patients were divided into high and low PSI groups based on the 80^th^ percentile. The below represented the splicing pattern indicated by the red line. **(E, F)** The correlation between the PSI index of MKNK2a/b isoforms and METTL3 expression in PCa, respectively. **(G)** The violin plot of PSI index of MKNK2a/b isoforms in METTL3 high and low groups, indicating increased expression of MKNK2a isoforms and decreased expression of MKNK2b isoforms in METTL3 high group. **(H)** The PSI index of the MKNK2 AS event (generating the MKNK2a isoform) in siMETTL3 or STM2457 (METTL3 inhibitor) C42 cells was reduced compared to the control group. **(I)** The top two panels represented the proportion of predicted m^6^A levels of MKNK2 in control (siCT) and METTTL3 knockdown (siM3) conditions. Y-axis represents the m^6^A modification percentage, which is the percent error of specific bases (%ESB) identified by ELIGOS2 software. The third and fourth panels represented the read coverage of m^6^A modification levels on the MKNK2 gene in normal prostate epithelial cells (RWPE-1) and PCa cells (LNCaP) from public sequencing data. Among them, the regions highlighted by four colors are regions with differential m^6^A modification levels in the exon 14a/b region of MKNK2. The bottom shows the structural features of MKNK2a/b isoforms. **(J)** The correlation between the PSI index of MKNK2a/b isoforms and 1-12 SRSFs expression in PCa, respectively. The red dot represented the significant correlation. **(K)** The correlation between the PSI index of MKNK2a/b isoforms and SRSF1 expression in PCa, respectively. siCT, siControl; siM3, siMETTL3; *p<0.05; **p<0.01; ***p <0.001; ****p<0.0001.

Based on previous functional enrichment analysis (**Figure 3B**), we noted that METTL3 was primarily associated with alternative splicing of genes linked to translation initiation. Among these genes, MNK2 (encoded by MKNK2) is the known kinase responsible for phosphorylating eukaryotic translation initiation factor 4E (eIF4E) at serine 209, an essential molecule during the mRNA translation phase (47). Therefore, we further interrogate whether the spliced event of MKNK2 that was validated in PCa cell lines was clinical significance. For MKNK2, two alternative isoforms were analyzed (namely MKNK2a and MKNK2b). The whole mRNA and protein levels of MKNK2 were evaluated in PCa, suggesting its onco-prognosis in PCa (**Figures 6A, B**). Moreover, we explored the relationship between MKNK2a/b isoforms and the prognosis of PCa patients. The data showed that a higher MKNK2a or a lower MKNK2b had a worse prognosis (PFI survival) (**Figures 6C, D**) and more biochemical recurrences (**Figure S12**), which indicated MKNK2 isoforms might play roles in PCa progression.

We further validated the relationship between METTL3 and MKNK2a/b isoforms using TCGA Spliceseq datasets. Correlation analysis showed that there was a significant positive/negative correlation between the expression of METTL3 and MKNK2a/b AS events (**Figures 6E, F**). Consistently, we observed that the AS events for MKNK2a displayed a significantly higher PSI value in METTL3 high patients than in the lower group, while the PSI values were significantly lower for MKNK2b (**Figure 6G**). These results suggest that METTL3 participated in the splicing process of the MKNK2 gene in PCa patients. Moreover, we also verified that METTL3 depletion (METTL3 inhibitor, STM2457) led to aberrant splicing of MKNK2 using two external datasets (**Figure 6H**) (48), which was also consistent with our previous findings. Collectively, these data indicated the role of METTL3 in modulating MKNK2 AS events in PCa.

The previous findings indicated that METTL3 regulates the splicing process associated with splicing factors and m^6^A sites. Our nanopore DRS data showed that after METTL3 knockdown, the m^6^A modification level in the MKNK2 exon 14a/b region was downregulated (**Figure 6I**). At the same time, we analyzed the public MeRIP-seq data and found that compared with normal prostate cells (RWPE-1), the exon 14a/b region of the MKNK2 gene in PCa cells has a higher methylation level (**Figure 6I**). Therefore, we speculate that the m^6^A modification in the exon 14a/b region of the MKNK2 gene may affect the binding of splicing factors to MKNK2. We next aimed to identify the potential splicing factors targeting MKNK2. Through the correlation test, we found that several SFs were positively correlated with the level of MKNK2a and negatively correlated with the MKNK2b level (**Figure 6J**). It’s known that the splicing process of MKNK2 pre-mRNA is regulated by SRSF1 (49). Here, we found a significant correlation between the expression of SRSF1 and MKNK2a/b isoforms in PCa (**Figure 6K**). However, RNA-Seq results showed that METTL3 knockdown did not affect the expression level of most SRSFs including SRSF1 (**Figure S13**). Previous studies indicated that m^6^A modification near splicing sites may affect the binding of splicing factors to transcripts. Therefore, we speculate that the m^6^A modification in the exon 14a/b region of the MKNK2 gene may affect the binding of splicing factors such as SRSF1 to MKNK2. The specific regulatory mechanism remains to be studied.

## 4 Discussion

Although mechanisms of METTL3 in many cancers have been well documented, the role of METTL3 in PCa-related AS switch has been poorly explored. Given the inherent differences in each cancer type, a tailored analysis focusing on PCa is still worth exploring and important for drug development. Our study illustrates some findings that are consistent with those in other cancers (e.g., the association between METTL3 and MYC signaling) (50–53), but we did find many PCa-specific findings that differ from other cancer types.

Prior research has found that high METTL3 expression can promote PCa cell proliferation, survival, and invasion *in vitro* and *in vivo* (9, 10, 54–56), however, our understanding of its role in AS regulation is still limited. Thus, we performed RNA-Seq to evaluate the function of METTL3 depletion in PCa. The results showed that METTL3 knockdown resulted in alterations in many cancers and cell proliferation-related genes, such as the FOXO signaling pathway, G2M Checkpoint, E2F Targets, and DNA repair hallmark. This was consistent with results in other cancers. In liver cancer, METTL3 can stabilize FOXO3 mRNA, reducing autophagy and sorafenib resistance (57). Moreover, the METTL3-METTL14 complex participates in DNA repair processing by aggregating at UV-exposed DNA damage sites (5, 58). Moreover, we also verified the expression changes of some genes by qPCR, which showed the same trend with RNA-Seq data and was in line with existing evidence. MSI1 is characterized as an RNA-banding protein (RBP) by repressing the mRNA translation and is associated with cancer stem cell properties of various cancers (59). A recent report has implicated that MSI1 regulated the expression of m^6^A reader YTHDF1, thus involving glioblastoma cell proliferation and migration (59). Interestingly, a preprinted study by Wei et al. indicated that IGF2BP3, another m^6^A reader, destabilized the m^6^A-harboring NF1 mRNA to accelerate triple-negative breast cancer progression, which was also consistent with our data (60). A similar reduction in NPEPL1 protein has been noted in another METTL3 knockdown proteomics dataset in LNCaP cells (39), the detailed mechanism between METTL3 and NPEPL1 in PCa remains unclear and needs to be explored.

We next focused on genome-wide METTL3-regulated AS events in PCa cell lines. Our results revealed that METTL3 primarily regulated the AS switch associated with cell cycle and translation initiation in PCa, and that AS regulation may be associated with MYC signaling. *C. Achour et al.* reported that METTL3 may indirectly regulate AS events through MYC in breast cancer (61). In addition, studies by *Yuan et al.* (9) and *C. Achour et al.* (61) also showed that METTL3 knockdown can suppress MYC protein levels, respectively, which is also common in several other cancers, such as AML, gastric cancer, colorectal cancer, and bladder cancer (50–53). These results suggest that the regulation of carcinogenesis by METTL3 through MYC is a common phenomenon and that METTL3 regulates AS events through a similar mechanism in prostate and breast cancer. In addition, we explored the association of AS alterations with the regulation of gene expression. However, our findings showed that only 9-15% of AS-affected genes overlapped with DEGs, with the majority being related to isoform-switched genes. This suggests that in PCa cells, AS dysregulation may have little effect on global gene expression but functionally tailored transcriptomes instead (43). Interestingly, we found that functional pathways enriched by AS genes (e.g., metabolic pathways, MYC, and E2F target markers) were coordinated with those in METTL3-affected DEGs. These results indicated that METTL3 jointly regulates PCa biological processes through multi-layered gene regulation, and has a broad and complex mechanism of action, which requires further in-depth study of its coordinated mechanism.

Notably, we found that after METTL3 depletion, two PCa cells had different regulatory responses on ES and IR events. Knockdown of METTL3 in AR^-^ cells (DU145) resulted in more introns being preserved and more exons being skipped, whereas the opposite phenomenon was observed in AR^+^ cells (LNCaP). It has been shown that m^6^A promotes exon inclusion or exon exclusion. A breast cancer study showed that depletion of METTL3 causes exons of specific genes to be retained (61). Conversely, another study showed that m^6^A promotes exon inclusion, and depletion of METTL3 leads to exon skipping (62). The differential effects of m^6^A on AS appear to be cell- or transcript-specific and depend on specific splicing factors. Studies have shown that m^6^A reader YTHDC1 binds to m^6^A sites and recruits the splicing factor SRSF3 to promote exon inclusion (15). A parallel study showed that the accumulation of m^6^A markers following FTO depletion in mouse preadipocytes promoted the binding of another splicing factor, SRSF2, leading to increased inclusion of target exons (16). Therefore, we also hypothesized that there is heterogeneity in the regulation of AS by METTL3 in AR^-^ or AR^+^ cells, depending on the cellular context and specific splicing factors. Exclusion of certain exons has been shown to be controlled by antagonism of the SRSF1/SRSF2 proteins, with SRSF1 inhibiting exon skipping and SRSF2 activating exon skipping (47). Our results showed that SRSF1 or SRSF2 were enriched in DU145 and LNCaP, respectively. These studies suggest that METTL3 may play different roles in RNA splicing in DU145 and LNCaP cells by interacting with different partners. However, the regulatory network of RNA splicing is complex and dynamic, and the mechanism of METTL3 in the two PCa cell lines requires more studies.

In this study, we took the MKNK2 gene as an example to explore the clinical significance of AS dysregulation in PCa. Our previous results showed that METTL3 mainly regulates alternative splicing of genes related to translation initiation. MNK1/2 are the only known kinases responsible for phosphorylating eukaryotic translation initiation factor 4E (eIF4E) at serine 209, which is a key rate-limiting molecule in the mRNA translation phase (63). Phosphorylation of eIF4E by MNKs proteins enhances its binding to the 5’cap structure of mRNA, thereby enhancing the cap-dependent translation process (64). MNK2/eIF4E signaling pathway is associated with the translation of tumorigenesis-associated mRNAs, contributing to cell proliferation, invasion, and drug resistance (49, 63, 65–69). Our results found that MKNK2 expression was higher in PCa patients than in normal samples. Moreover, splicing variants of MKNK2a were significantly associated with poor PFI in PCa patients and significantly positively correlated with METTL3 expression. These results suggest that METTL3 was also associated with AS of MKNK2 in PCa clinical samples, and the MKNK2a subtype may play a role in promoting cancer progression. At the same time, we also verified that METTL3 knockdown led to abnormal splicing of MKNK2 and reduced expression of MKNK2a isoforms using full-length transcriptome and qPCR. Therefore, METTL3 may affect the translation process of oncogenes by regulating the AS of the translation initiation-related gene MKNK2.

Finally, we explored the possible mechanism of METTL3 in regulating MKNK2 splicing. The variable splicing process of genes is controlled by a variety of regulatory elements, mainly including splicing factors and splicing sites (42). It is known that MKNK2 pre-mRNA is one of the target molecules of the splicing factor SRSF1 (49), which is proven to modulate the MKNK2a-MKNK2b isoforms switch. In our data, a significant correlation was found between the expression of SRSF1 and MKNK2a/b isoforms in PCa, which is consistent with the literature. Furthermore, the binding motif of SRSF1 was significantly enriched in METTL3-regulated splicing events. These results suggest that SRSF1 may be involved in the regulation of MKNK2 alternative splicing by METTL3. However, RNA-Seq results showed that METTL3 knockdown did not affect the expression level of SRSF1. Existing studies have shown that m^6^A modification near the cleavage site can affect the binding of cleavage factors to transcripts, thereby changing the way the transcripts are cleaved. Our results also showed that m^6^A sites overlap significantly with splice junctions, suggesting that m^6^A is involved in the regulation of splice site recognition. Further, the data revealed that the methylation level of the exon14a/b region of the MKNK2 gene in PCa cells was significantly higher than that in normal cells. And after METTL3 knockdown, the predicted methylation level of exon exon14a/b region was significantly reduced. Therefore, the declining m^6^A level in MKNK2 caused by METTL3 knockdown potentially reduces the binding of splicing factors. In the future, further experiments are needed to verify the mechanism of METTL3 regulating MKNK2 AS events.

This study has some limitations. First, while offering advantages such as long read lengths and RNA modification analysis, the DRS approach has limitations of higher error rates and more complex bioinformatics analysis. Our results are mainly based on bioinformatic analysis, and further research in experimental cohorts is warranted. It is worth mentioning that our conclusions are supported by other independent datasets and qPCR experiments, demonstrating the validity. Second, we uncovered an intriguing mechanism of METTL3 in AR^-^ and AR^+^ PCa cells. However, which factors are functionally related to these responses remains unanswered, which may enhance the understanding of the carcinogenesis process of different PCa stages.

In summary, our study used genome-wide analyses to investigate METTL3-regulated AS events in AR^+^ and AR^-^ PCa cell lines via a combination of standard RNA-Seq and long-reads direct RNA-Seq of Nanopore. We revealed both common and phenotypic heterogeneity of different PCa cells regulated by METTL3, which were potentially associated with m^6^A modification and SFs. The study identified the clinical relevance of MKNK2 AS events in PCa and explored the potential mechanism of METTL3 in modulating MKNK2 AS events. These findings provide new insights into RNA modifications in PCa and could serve as a favorable molecular basis for novel treatment strategies in androgen-sensitive and androgen-resistant prostate cancer.

## Data availability statement

The datasets presented in this study can be found in online repositories. The names of the repository/repositories and accession number(s) can be found in the article/**Supplementary Material**.

## Ethics statement

Ethical approval was not required for the studies on humans in accordance with the local legislation and institutional requirements because only commercially available established cell lines were used.

## Author contributions

LW: Study design, Data processing, and Writing-Original draft preparation. LYW, YW, and CF: discussed the data and assisted in the sequencing library preparation. LS, YL, JN and CY: validated the differentially expressed or spliced genes. JH, PC, and CN: Writing-Reviewing and Editing. ST: Supervision, Writing-Reviewing and Editing. All authors contributed to the article and approved the submitted version.
