# Supplementary material for: Dissecting the effects of METTL3 on alternative splicing in prostate cancer
Source: Front Oncol. 2023 Aug 22;13:1227016. doi: 10.3389/fonc.2023.1227016 (PMC10477979; doi:10.3389/fonc.2023.1227016)
Supplement: Supplementary file 1 [file DataSheet_1.docx]

Supplementary Material

Dissecting the effects of METTL3 on alternative splicing in prostate cancer

**Lin Wang^1,2^, Ling Shi^2^, Yonghao Liang^2^, Judy Kin-Wing Ng^2^, Chan Hoi Yin^2^, Lingyi Wang^2^, Jinpao Hou^2^, Yiwei Wang^2^, Cathy Sin-Hang Fung^2^, Peter Ka-Fung Chiu^3^, Chi-Fai Ng^3^ and Stephen Kwok-Wing Tsui^2,4*^**

*** Correspondence:** Prof. Stephen Kwok-Wing Tsui, kwtsui@cuhk.edu.hk

# Supplementary Figures

## Supplementary Figures


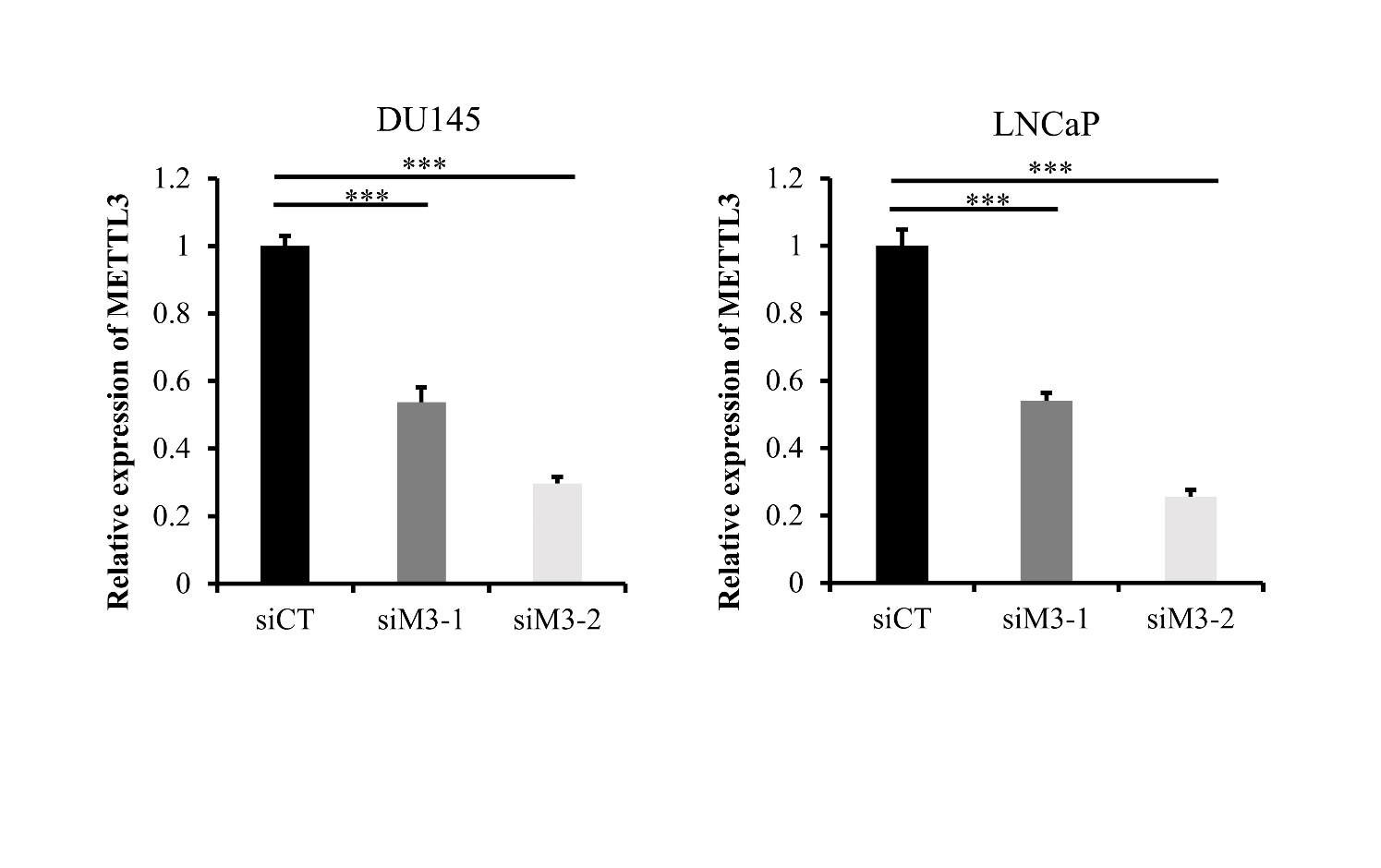


**Figure S1.** Expression of METTL3 in DU145 and LNCaP cells treated with siControl or two siRNA against METTL3. The bar plot showed the average relative expression of METTL3 normalized against GAPDH of triplicate samples from three individual experiments. siM3-1 and siM3-2, represent two siRNA against METTL3. ***, P≤0.001.


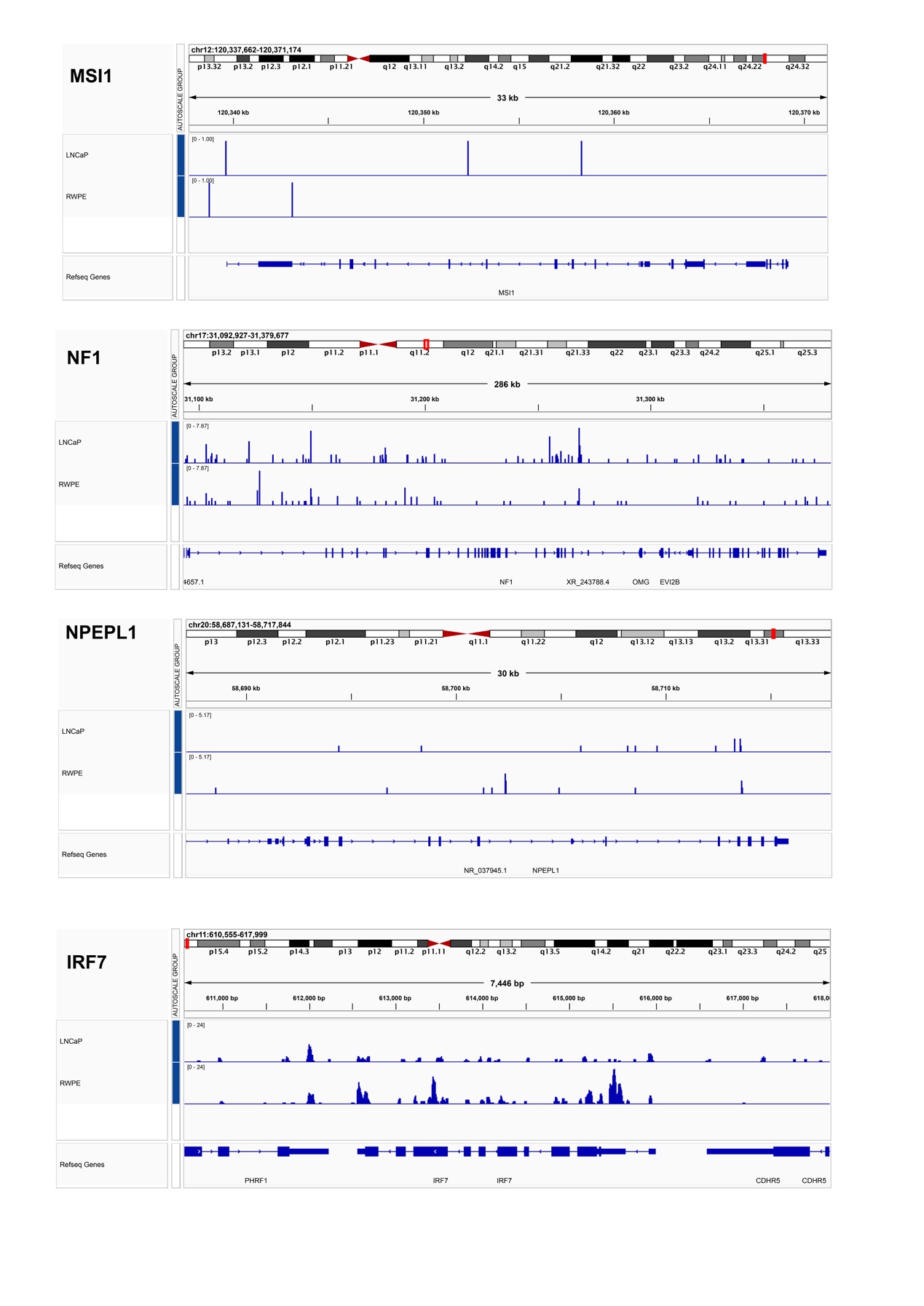


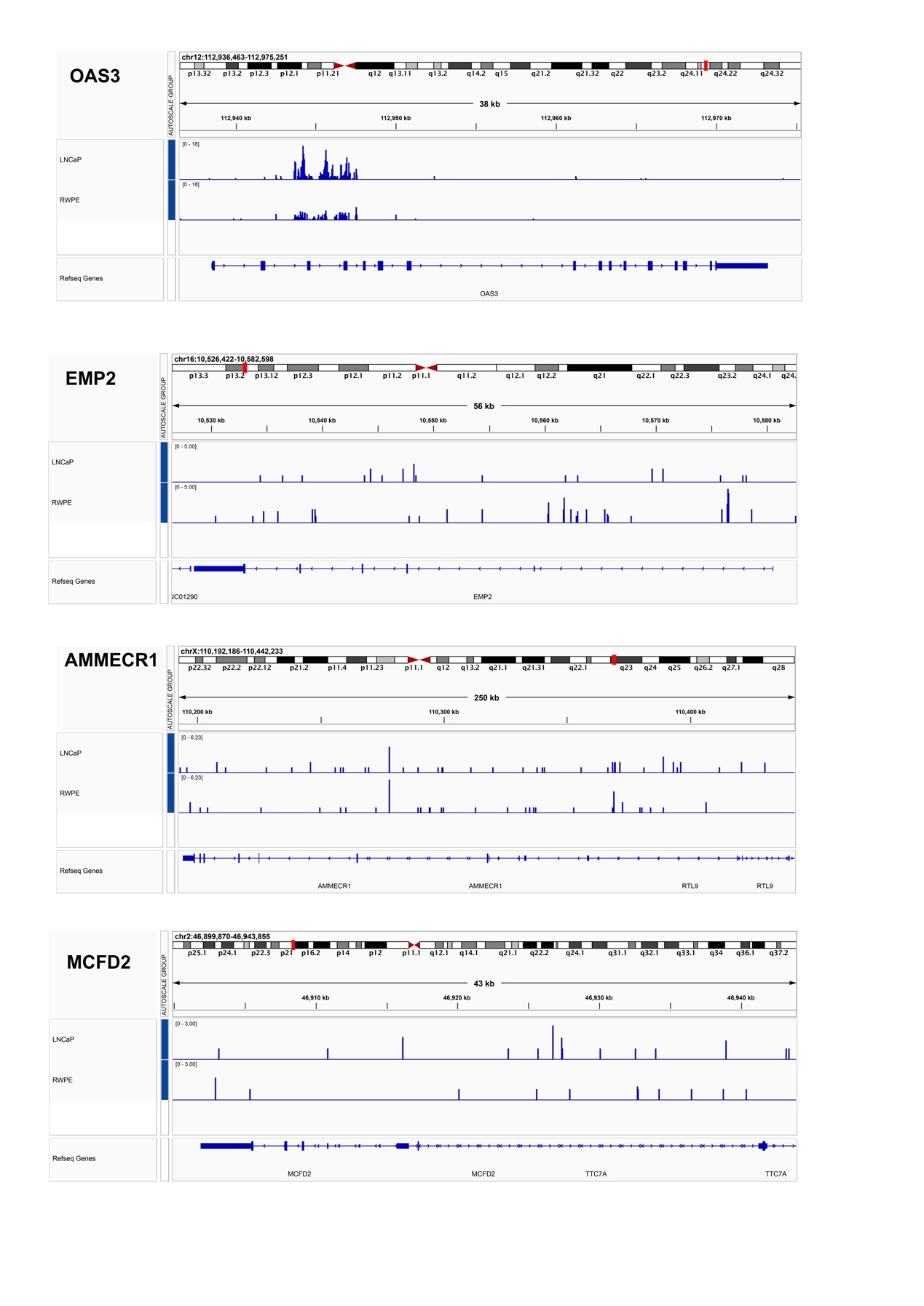


**Figure S2.** The read coverage of m^6^A modifications in eight DEG genes. The IGV screenshots show the read coverage of m^6^A modifications in both prostate cancer cells (LNCaP, upper) and normal prostate cells (RWPE-1, lower), which was retrieved from the dataset GSE161303 identified by miCLIP.


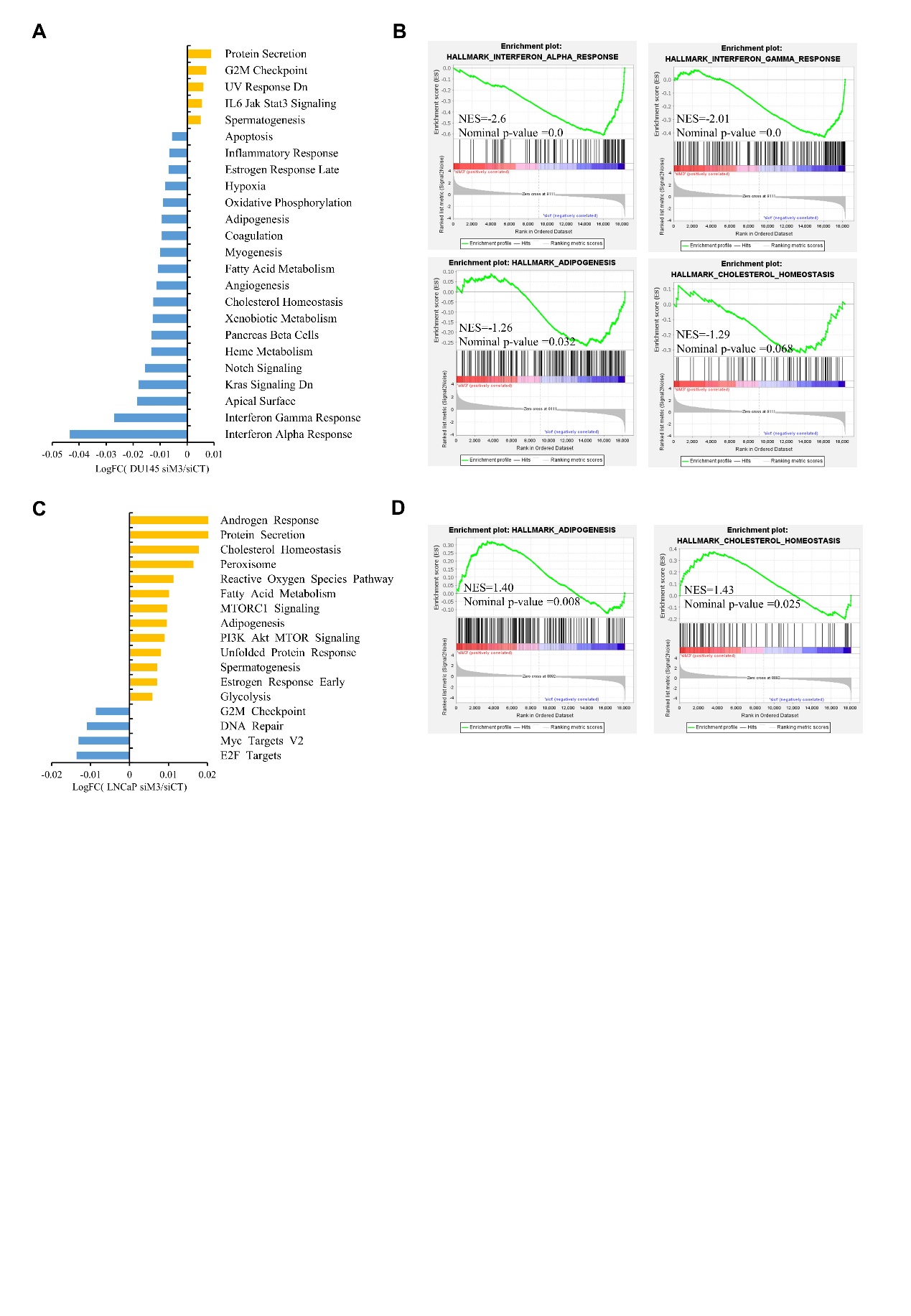


**Figure S3.** GSVA analysis of DEGs of the two PCa cell lines. (A and C) Bar plot showing the enrichment of cancer hallmarks from MsigDB using GSVA in DU145 and LNCaP cells, respectively. (B-D) GSEA enrichment results of several specific pathways in DU145 and LNCaP cells, respectively. siM3, siMETTL3; siCT, siControl.


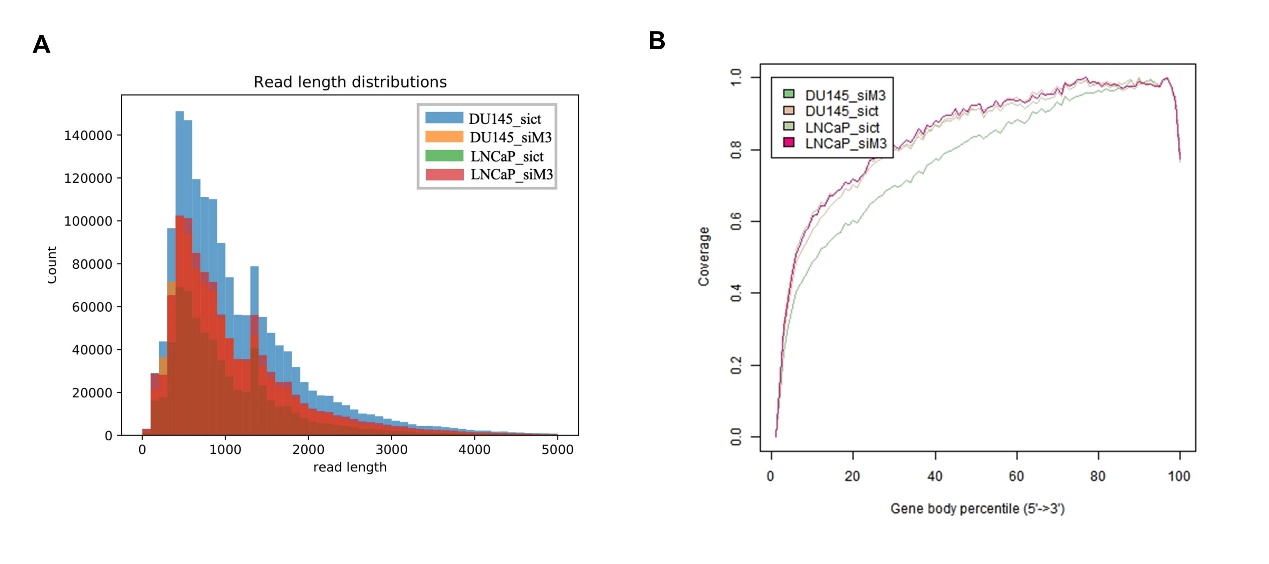


**Figure S4.** Statistics summary of direct RNA sequencing. (A) The length distribution of passed reads in each sequenced sample. (B) Gene body coverage of each sequenced sample. Length of all genes normalized to 100. 5’ end of the gene on the left-hand side (0) to 3’ end on the right-hand side (100). Lines show mean coverage across all genes across the length of the gene body.


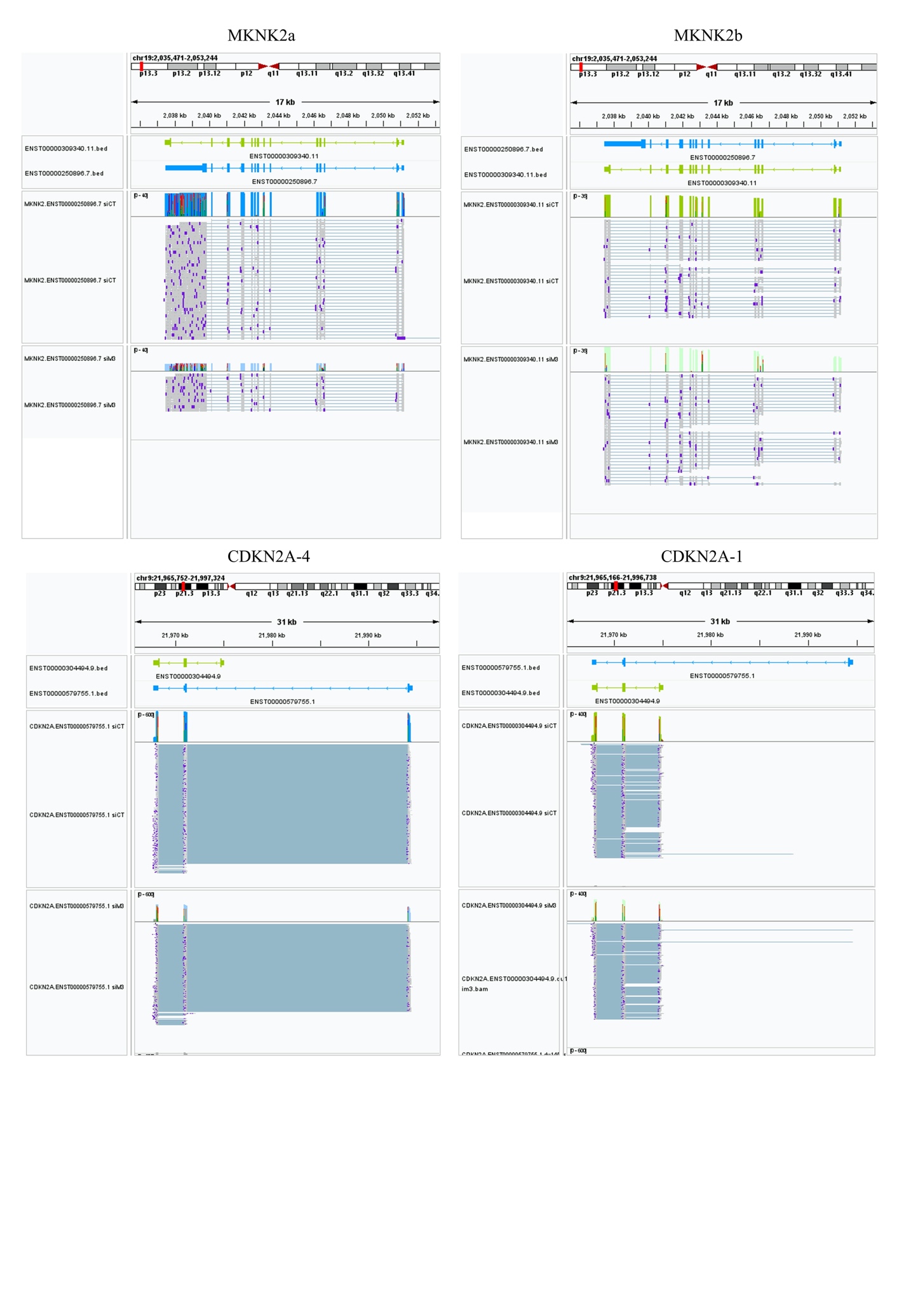


**Figure S5.** Read coverage of two main transcript isoforms from MKNK2 and CDKN2A genes. The top panel of IGV screenshots displayed the read coverage of MKNK2a and MKNK2b isoforms under control and METTL3 knockdown conditions of DU145 cells. The bottom panel indicated the read coverage of CDKN2A-4 and CDKN2A-1 isoforms under control and METTL3 knockdown conditions of DU145 cells. The mixed color bar indicated the proportion of A, T, C, and G at this site (A= Green, T=Red, C=Blue, G=Brown), supposing base-calling errors at this site.


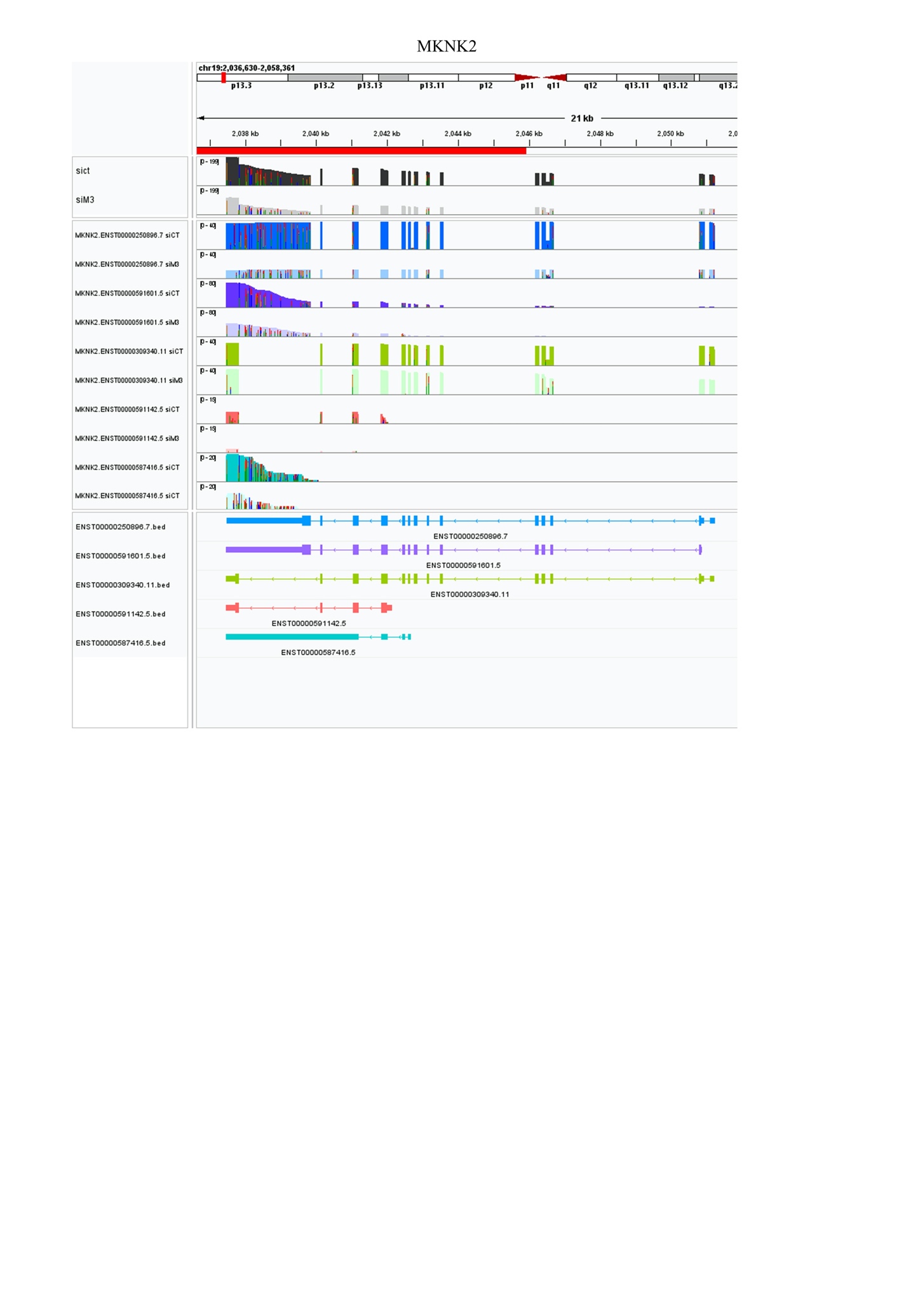


**Figure S6.** Read coverage of detected transcript isoforms of MKNK2 genes in DU145 cells. The top two panels of IGV screenshots depicted the read coverage of the MKNK2 gene under control and METTL3 knockdown conditions. Subsequent panels represented the read coverage of five transcript isoforms in control and knockdown groups, and each label with a different color. The bottom panel illustrated the genomic structure of these five isoforms. The mixed color bar indicated the proportion of A, T, C, and G at this site (A= Green, T=Red, C=Blue, G=Brown), supposing base-calling errors at this site.


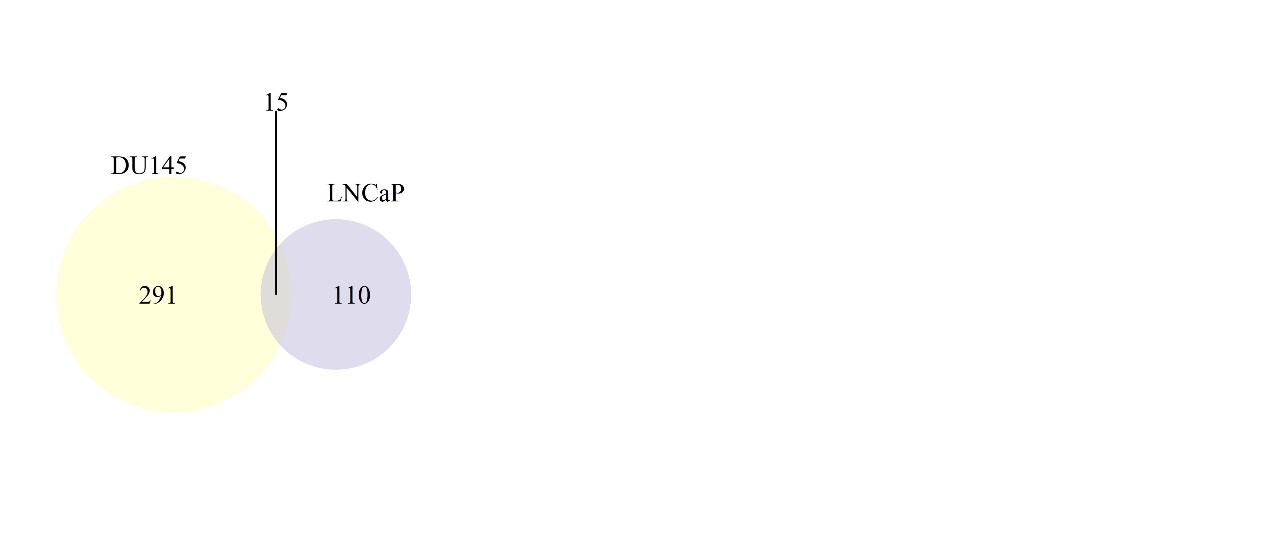


**Figure S7.** The overlap of AS-related genes between DU145 and LNCaP cells. (A) Venn plot showed the overlapped AS-related genes between DU145 and LNCaP cells.


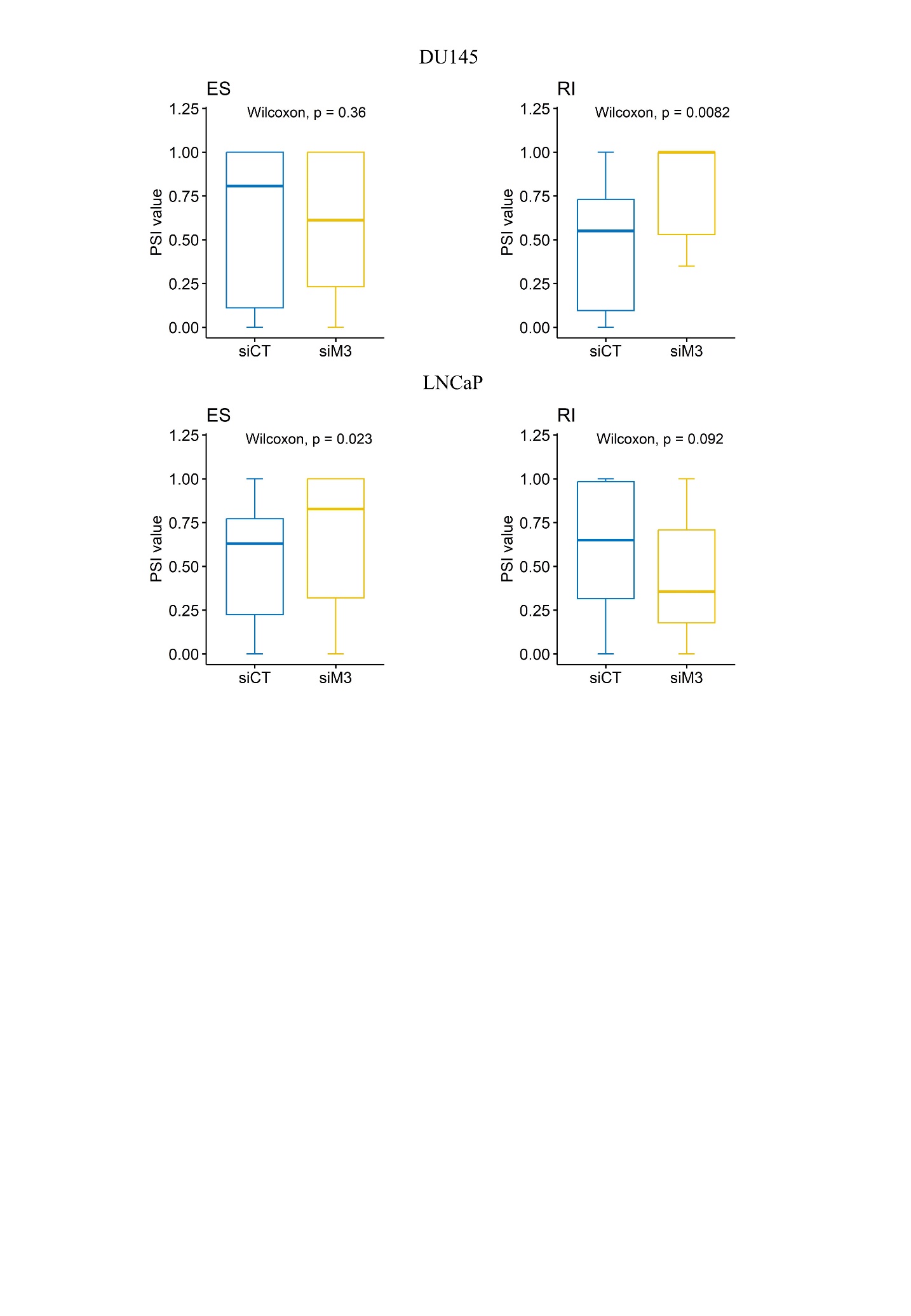


**Figure S8.** The difference in PSI distributions between control and knockdown groups. Box plots showing PSI values of differential IR and ES events (FDR<0.05) in DU145 and LNCaP cells, respectively.


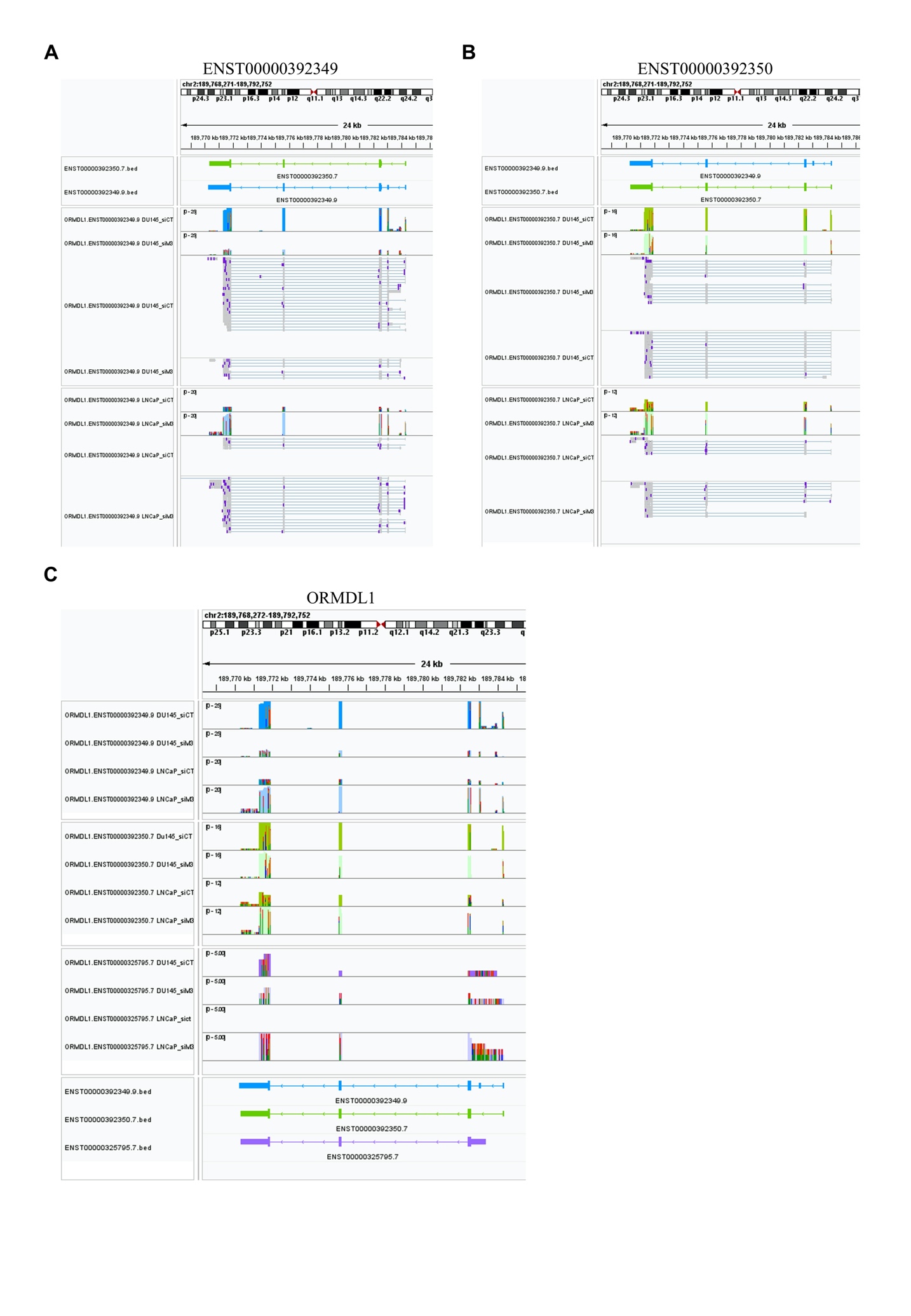


**Figure S9.** Read coverage of two main isoforms of the ORMDL1 gene. A. IGV screenshots illustrated the genomic structure of isoform 1 (ENST00000392349) (Panel one) and its read coverage under control and knockdown conditions in DU145 (Panel two) and LNCaP (Panel three) cells. B. IGV screenshots illustrated the genomic structure of isoform 2 (ENST00000392350) and its read coverage under control and knockdown conditions in DU145 (Panel two) and LNCaP cells (Panel three). C. Read coverage of three detected transcript isoforms of ORMDL1 genes in DU145 and LNCaP cells, namely isoform 1 (ENST00000392349), isoform2 (ENST00000392350), and isoform 3 (ENST00000325795). The bottom panel illustrated the genomic structure of these three isoforms.


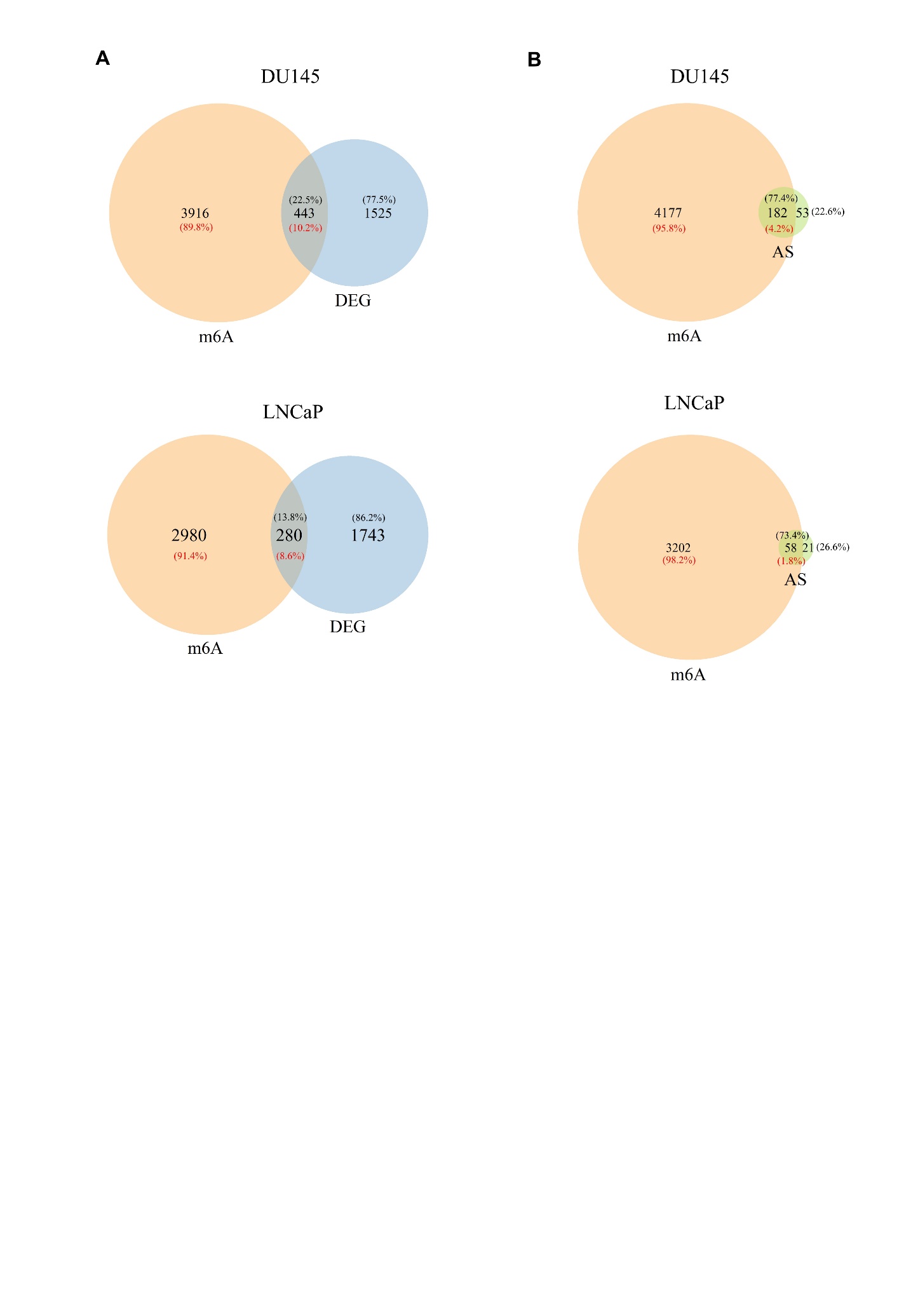


**Figure S10.** The intersection between m^6^A-modified genes and DEGs or AS-related genes in DU145 and LNCaP cells. (A) Venn plot showed the overlap between m^6^A-modified genes and DEGs in DU145 (top) and LNCaP (bottom) cells. (B) Venn plot showed the overlap of m^6^A-modified genes and AS-related genes in DU145 (top) and LNCaP (bottom) cells. The percentage count in red indicates the proportion of m^6^A-modified DEGs or m^6^A-modified AS genes out of the total m^6^A-modified genes. The percentage count in black represents the proportion of m^6^A-modified DEGs or m^6^A-modified AS genes out of the total DEG or AS genes.


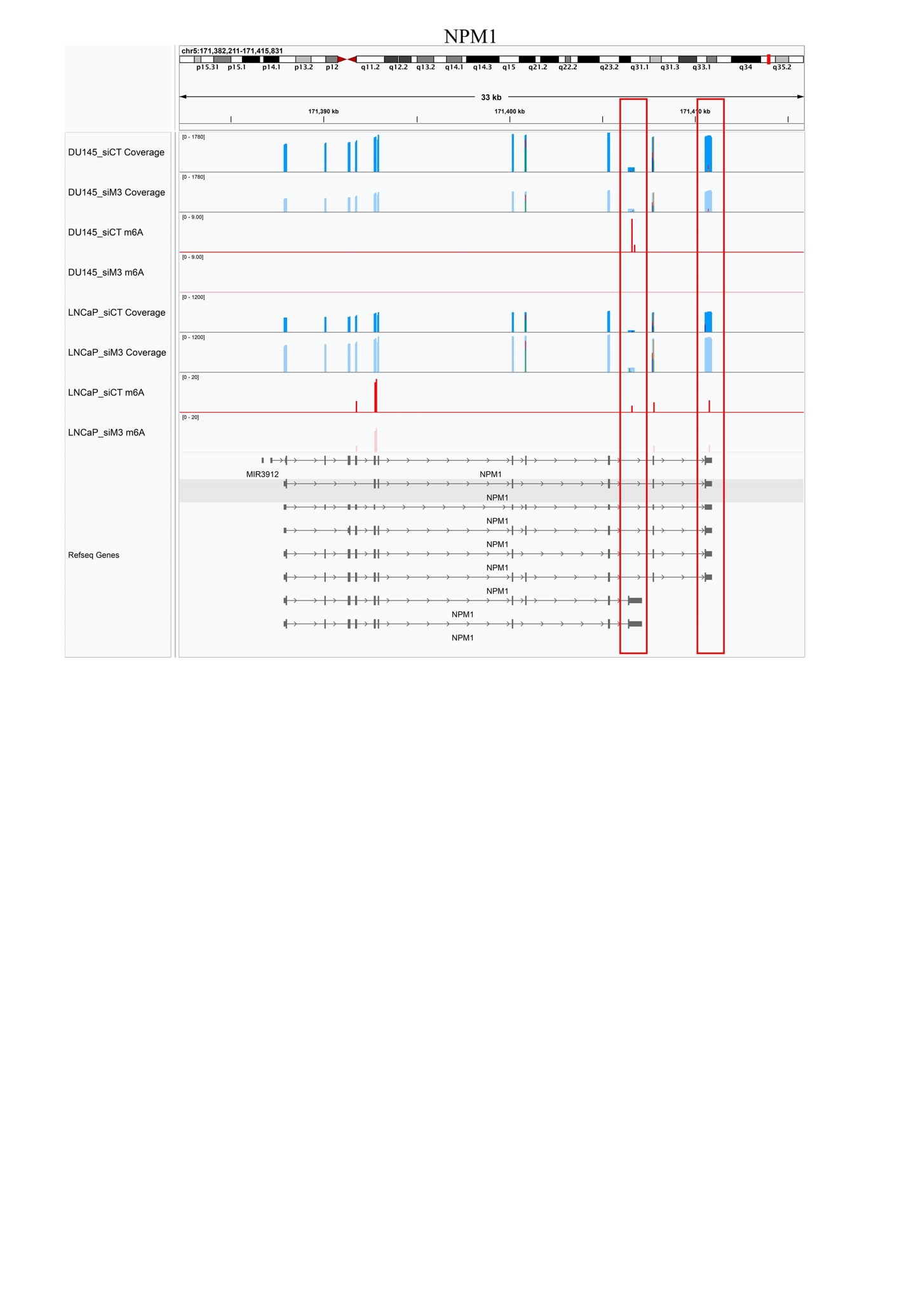


**Figure S11.** Read coverage and m^6^A levels of NPM1 isoforms. The top four panels of IGV screenshots illustrated the read coverage and m^6^A levels of NPM1 isoforms under control and knockdown conditions of DU145 cells. The following four panels showed the read coverage and m^6^A levels of NPM1 isoforms under control and knockdown conditions of LNCaP cells. The bottom section displayed the genomic structure of all NPM1 isoforms. The red box highlighted the differential m^6^A sites observed in distinct isoforms.


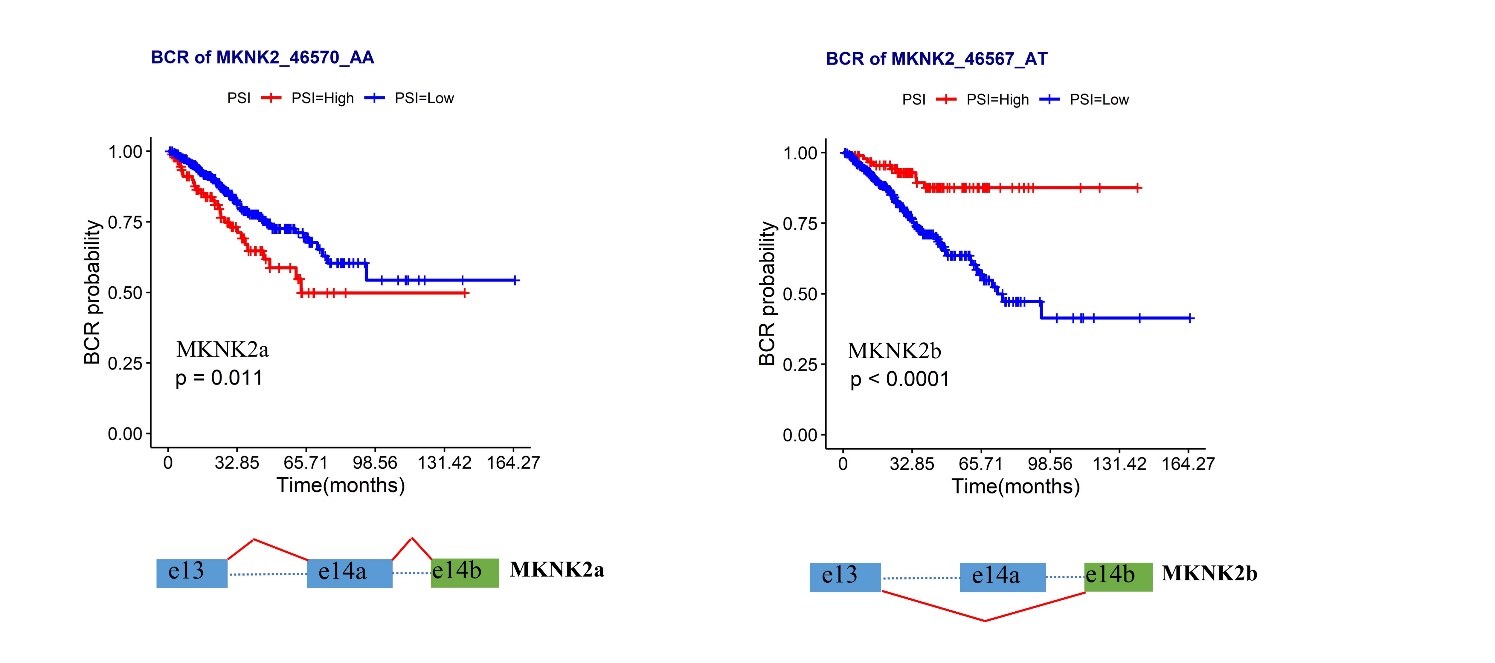


**Figure S12.** Clinical significance of MKNK2 isoforms in prostate cancer. Survival analysis of biochemical recurrence (BCR) showed a significant positive/negative correlation between MKNK2a/b isoforms and a higher recurrence in PCa patients. The below represented the splicing pattern indicated by the red line. Patients were divided into high and low PSI groups based on the 80^th^ percentile.


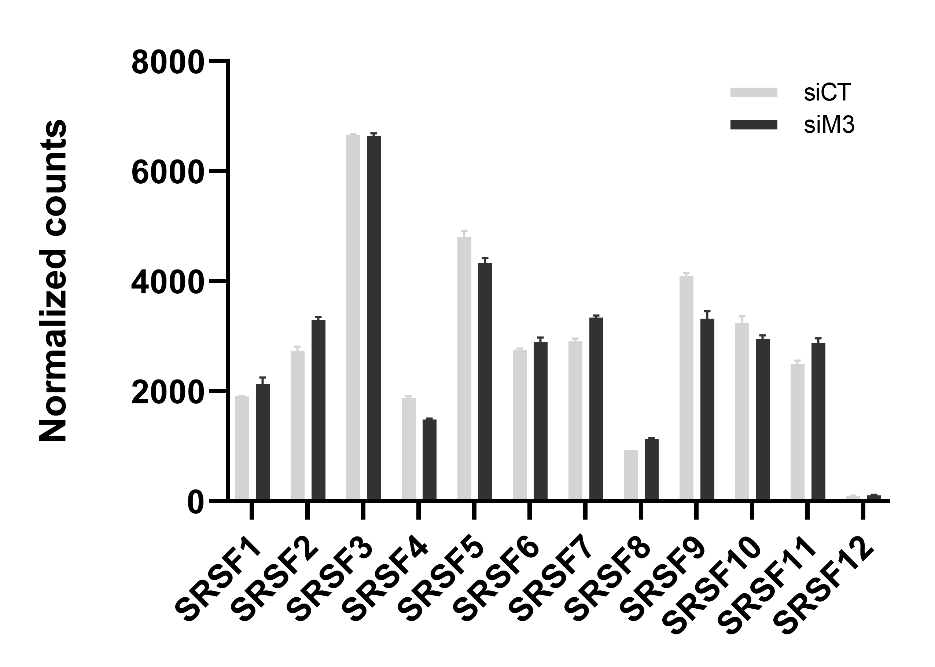


**Figure S13.** The expression level of 1-12 SRSFs in control and siMETTL3 group of DU145 cells. siCT, siControl; siM3, siMETTL3.
